# Diminished Vision in Healthy Aging Is Associated with Increased Retinal L-Type Voltage Gated Calcium Channel Ion Influx

**DOI:** 10.1371/journal.pone.0056340

**Published:** 2013-02-14

**Authors:** David Bissig, Dennis Goebel, Bruce A. Berkowitz

**Affiliations:** 1 Department of Anatomy and Cell Biology, Wayne State University, Detroit, Michigan, United States of America; 2 Department of Ophthalmology, Wayne State University, Detroit, Michigan, United States of America; Dalhousie University, Canada

## Abstract

Extensive evidence implicates an increase in hippocampal L-type voltage-gated calcium channel (L-VGCC) expression, and ion influx through these channels, in age-related cognitive declines. Here, we ask if this “calcium hypothesis" applies to the neuroretina: Is increased influx via L-VGCCs related to the well-documented but poorly-understood vision declines in healthy aging? In Long-Evans rats we find a significant age-related increase in ion flux through retinal L-VGCCs *in vivo* (manganese-enhanced MRI (MEMRI)) that are longitudinally linked with progressive vision declines (optokinetic tracking). Importantly, the degree of retinal Mn^2+^ uptake early in adulthood significantly predicted later visual contrast sensitivity declines. Furthermore, as in the aging hippocampus, retinal expression of a drug-insensitive L-VGCC isoform (α_1D_) increased – a pattern confirmed *in vivo* by an age-related decline in sensitivity to L-VGCC blockade. These data highlight mechanistic similarities between retinal and hippocampal aging, and raise the possibility of new treatment targets for minimizing vision loss during healthy aging.

## Introduction

Extensive research in the CA1 region of the rat hippocampus has revealed an age-related increase in neuronal Ca^2+^ influx though L-type voltage-gated calcium channels (L-VGCCs) that is strongly linked with impaired synaptic plasticity and reduced cognitive function [1]–[5]. Diminished visual performance is another important behaviorally-evident functional decline that occurs with aging, beginning in young adulthood, but whose underlying mechanisms are poorly understood [6], [7]. Concurrent declines in neuroretinal function, when measured by electroretinogram (ERG), have also been noted: rod sensitivity and the maximum amplitude of rod responses to light both decrease with age [8], [9]. However, as reviewed by Spear [10], such physiological changes were too modest to account for the age-related vision declines. Here, we test an alternative hypothesis: that changes in retinal ion influx via L-VGCCs occur with age, and are linked to visual performance declines.

To test this hypothesis, we longitudinally and non-invasively measured the extent of retinal ion influx via L-VGCCs in light and dark-adapted retinas using Mn^2+^-enhanced MRI (MEMRI). In MEMRI, awake and freely-moving animals are injected with a non-toxic dose of the MRI contrast agent Mn^2+^, a Ca^2+^ surrogate that accumulates in neurons over a period of a few hours. Later, the animal is anesthetized and extent of retinal Mn^2+^ uptake is measured using MRI. Importantly, similar to Ca^2+^, Mn^2+^ primarily enters neurons through L-VGCCs: *In vitro*, Mn^2+^ uptake is strongly inhibited by L-VGCC blockers, and increased both by membrane depolarization (opening L-VGCCs) and the L-VGCC agonist BayK8644 [11], [12]. *In vivo* studies confirm that Mn^2+^ uptake is inhibited by the L-VGCC antagonists verapamil [13], nifedipine [14], and diltiazem [15]. Because Mn^2+^ efflux is slow, taking days to leave the retina [16], uptake measured a few hours after injection is a useful measure of ion influx through L-VGCCs.

In this study, two groups of rats were examined longitudinally – one from young to mid-adulthood, and the other from mid- through old adulthood. MEMRI data were compared to two aspects of visual performance; spatial frequency threshold (‘SFT’; a proxy for visual acuity) and contrast sensitivity (‘CS’). We measured visual performance using optokinetic tracking (OKT), a reflex which requires no animal training and avoids the potential confound of age-related impairments in thermoregulatory function [17]. In both groups, the first and final vision tests were followed by MEMRI: Using an eye patch to keep one eye dark-adapted while the other was exposed to normal lab lighting, we tested for longitudinal changes in retinal ion influx in both lighting conditions. Dark-adapted values represent maximal ion influx in outer retina, which is populated almost exclusively by photoreceptors, while subtracting light from dark values provides quantification of activity-dependent photoreceptor Mn^2+^ uptake. Anterior to the outer retina resides the inner retina, a brain-like complex of bipolar, amacrine, and ganglion cells that process light information gathered by the photoreceptors. Age-related ion-regulatory changes may also occur in the inner retina. However, the spatial overlap of light-activated and light-inactivated neurons makes unraveling the relationship between activity and Mn^2+^ uptake in inner retina more difficult than in outer retina. We therefore provide inner retinal data as Supplemental Material, while focusing on the photoreceptor-dominated outer retina to test our hypothesis that changes in retinal ion influx via L-VGCCs occur with age.

In a separate set of experiments, we verified that retinal Mn^2+^ uptake was inhibited by both topical and systemic L-VGCC blockade. Next, we used Western blots to assess age-related changes in retinal L-VGCC expression, expecting an age-related increase for only the α_1D_ isoform, based on findings from CA1 of the rat hippocampus [18], [19]. Because α_1D_ L-VGCCs are roughly an order of magnitude less-sensitive to antagonism than the α_1C_ isoform [20]–[22], we also used MEMRI to non-invasively test for an age-related loss in sensitivity to L-VGCC blockade.

## Results

### Visual Performance

**Figure 1:**
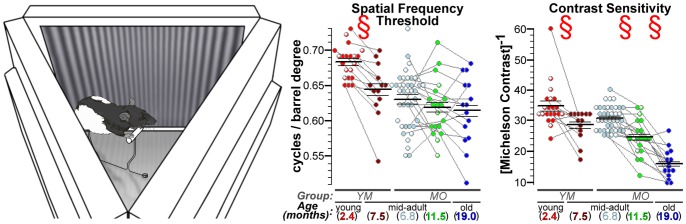
Visual performance declines with age. Spatial frequency threshold (SFT) and contrast sensitivity (CS) measurements were performed on an optokinetic tracking (OKT) device built in-house. *Left:* An oblique view of the OKT device, with the mirrored ceiling removed to show the typical position of a compliant rat: standing on an elevated perch, with its head roughly centered in the device. Movement was unrestricted during testing, and rats were gently placed back on the perch if needed. Tracking was recorded only if the rat remained on the perch *and* the stimulated eye (left eye if the virtual barrel was moving clockwise; right if counter-clockwise) was inside a small central portion of the device (see Fig. S1 in File S1). Additional device details – including illustrations, dimensions, reproducibility of measurements, and comparisons to a commercially-available system [47] – are provided as Supplemental Figure S1 (in File S1). *Center and Right:* Scatter plots showing longitudinal changes in SFT and CS. Spatial frequency of the stimulus is reported by “cycles/barrel degree” (c/bd) such that a (40/360° = ) 0.111 c/bd setting means that the three screens, together, generated a 360° virtual barrel with 40 dark-light-dark cycles of the sine-wave grating. Mean±SEM are indicated by the horizontal lines overlaid on each scatter plot. Longitudinal measures from the same subject are connected by gray lines, while a white mark within a point denotes the lack of follow-up data (e.g., due to animal death). Note that values from rats lost to follow-up are evenly distributed among those from animals retained for longitudinal comparisons. § indicates a significant (q<0.05; paired two-tailed tests) effect of age. Comparisons of ∼7 to ∼19 mo data are not labeled since patterns of significance (SFT: *P = *0.57, CS: *P = *2.1e-7) are well-depicted by the ∼7 vs. ∼11.5 mo comparisons. SFT tends to stabilize after the initial decline from young to mid-adulthood. CS progressively declines with age.

We first characterized longitudinal changes in visual performance in Long Evans rats using OKT, which is based on the reflexive head-movements of awake and free-moving rats in the presence of visible stimuli. SFT declined from *Y*oung to *M*id-adulthood (Group *YM*; *P = *2.4e-4) but remained stable from *M*id- to *O*ld adulthood (*P*>0.26 for all comparisons within Group *MO*). In contrast, CS declined significantly with age in both Group YM (*P = *3.4e-2) and Group MO (*P = *2.7e-4 for ∼7 to ∼11.5 mo; *P = *1.3e-4 for ∼11.5 to ∼19 mo) (Fig. 1). These results confirm and extend prior findings of visual performance declines with aging in other rodents [23], [24].

### Outer Retinal Mn^2+^ Uptake Changes with Age

**Figure 2:**
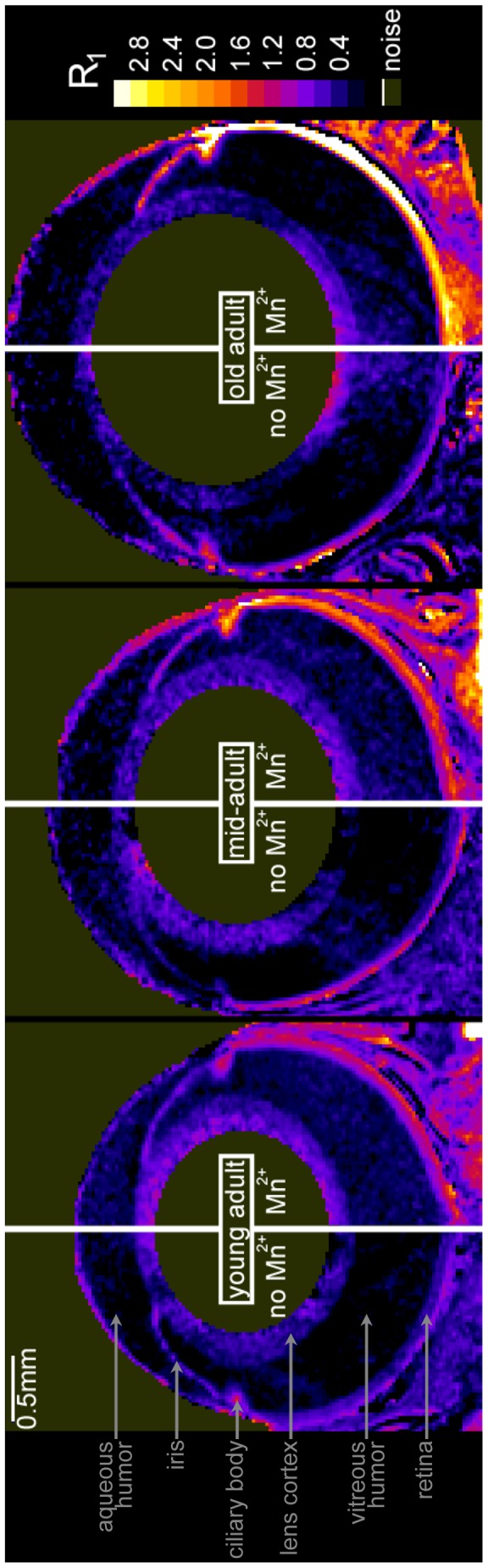
Outer retinal Mn^2+^ uptake visibly increases with age. Representative maps of tissue R_1_ (in units of s^−1^) from baseline (no Mn^2+^) and Mn^2+^-injected rats. In Mn^2+^-injected rats, R_1_ – which is linearly related to tissue Mn^2+^ concentration [58]– increases with age. As detailed in Supplemental Figure S2 (in File S1), this change could not be attributed to altered integrity of the blood-retinal barrier, which was fully intact at all ages. There was no relationship between age and baseline R_1_s in dark-adapted or light-exposed retinas, nor between age and dark-light differences in R_1_ (−0.18<r<0.17 with *P*>0.66; in dark, mean±SEM baseline R_1_s were 0.64±0.01, 0.49±0.05, and 0.59±0.04 s^−1^ respectively in young, mid-, and old adults; in light, 0.59±0.05, 0.50±0.03, and 0.60±0.03 s^−1^ respectively). Given that baseline R_1_s were stable, the average for outer retina (0.568 s^−1^) was subtracted from Mn^2+^-injected R_1_s to calculate ΔR_1_s – which are directly proportional to Mn^2+^ uptake – shown in Figure 3. Note that these R_1_ maps are for illustration only: Formal measurements used linearized and spatial-normalized retinal data. Also note that these R_1_ maps are sub-optimal for displaying the fine structural detail (layering) of the retina, which we routinely observe at the present image resolution (see Fig. S3 in File S1).

**Figure 3:**
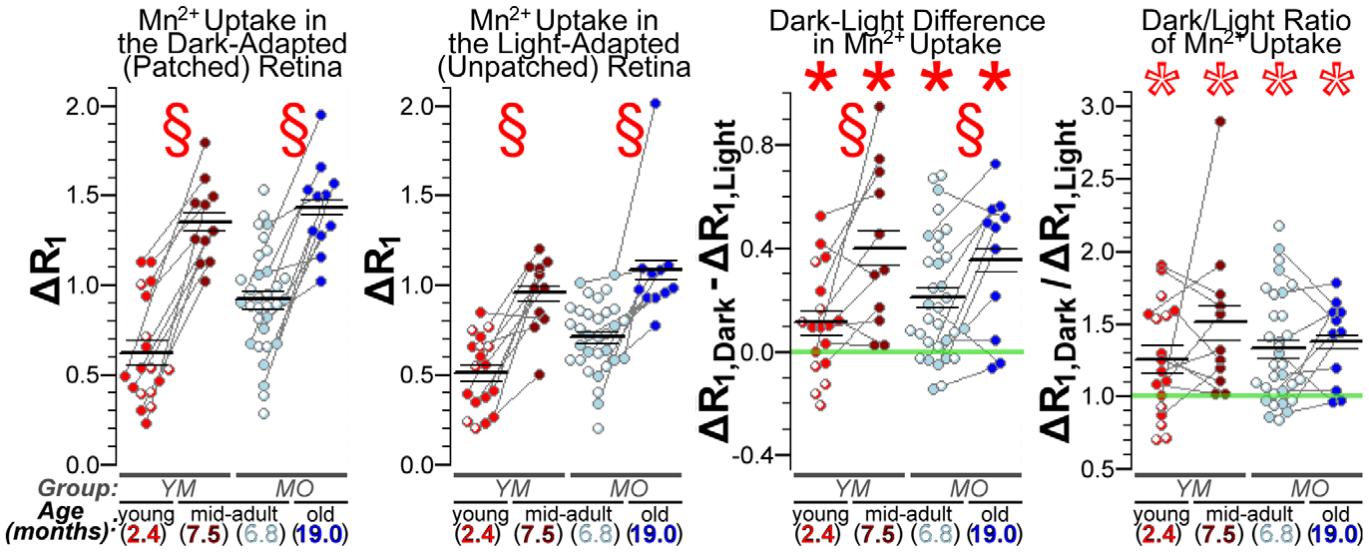
Quantification of age-related increase in outer retinal Mn^2+^ uptake. Scatter plots showing outer retinal ΔR_1_ (in s^−1^) from each Mn^2+^-injected rat. § indicates a significant (q<0.05; paired two-tailed tests) effect of age in longitudinal comparisons – young vs. mid-adults in Group YM, and mid- vs. old adults in Group MO. Retinal Mn^2+^ uptake (ΔR_1_s) increased significantly with age in each group, in both darkness and light. Comparing light and dark data, *’s denote significantly greater Mn^2+^ uptake in dark than light (filled *’s for dark-light differences >0, all *P*<0.018; open *s for dark/light ratios >1, all *P*<5.8e-3). Dark-light differences increased with age (q<0.05) but, interestingly, the dark-to-light ratio of Mn^2+^ uptake did not (both *P*>0.4). Age-related changes in inner retinal Mn^2+^ uptake and other MRI measurements (e.g. eye morphology) are provided in Supplemental Figure S3 (in File S1).

Next, we quantified retinal ion influx *in vivo* using MEMRI. Dramatic age-related increases in retinal Mn^2+^ uptake were easily visualized against the stable baseline (i.e., no Mn^2+^) R_1_ (Fig. 2). Subtracting baseline from Mn^2+^-enhanced data allowed for quantitative examination of tissue ΔR_1_s, which are directly proportional to tissue Mn^2+^ concentration. We found significant age-related increases in outer retinal Mn^2+^ uptake between young and mid-adulthood (Group YM; dark: *P* = 3.4e-5; light: *P* = 1.9e-4), and between mid- and old adulthood (Group MO; dark: *P* = 1.7e-4; light: *P* = 3.8e-4) (Fig. 3). These age-related increases in Mn^2+^ uptake were observed in both light-exposed and dark-adapted (patched) eyes. The inner retina showed similar changes (see Supplemental Figure S3 in File S1).

The expected pattern of activity-dependent outer retinal Mn^2+^ influx – low in photoreceptors exposed to light, when membranes are hyperpolarized and L-VGCCs are closed, but high in darkness, when photoreceptors are fully depolarized (for review, see Yau, 1994) – was noted at all ages in our longitudinal studies: Dark-light differences in outer retinal Mn^2+^ uptake were significantly greater than zero (all *P*<0.018), and dark/light ratios were significantly greater than 1 (all *P*<5.8e-3) (Fig. 3). The absolute amount of activity-dependent Mn^2+^ uptake (i.e., dark-light differences in ΔR_1_) increased significantly with age – both from young to mid- adulthood (Group YM; *P* = 0.038), and mid- to old adulthood (Group MO; *P* = 0.027) – but, interestingly, the relative amount (dark/light ratio) did not (*P*>0.40 in both groups) (Fig. 3). These data suggest that, despite some consistencies in retinal light responses across age groups, there is a robust age-related increase in net ion influx through photoreceptor L-VGCCs.

### Outer Retinal Mn^2+^ Uptake Predicts CS Declines

**Figure 4:**
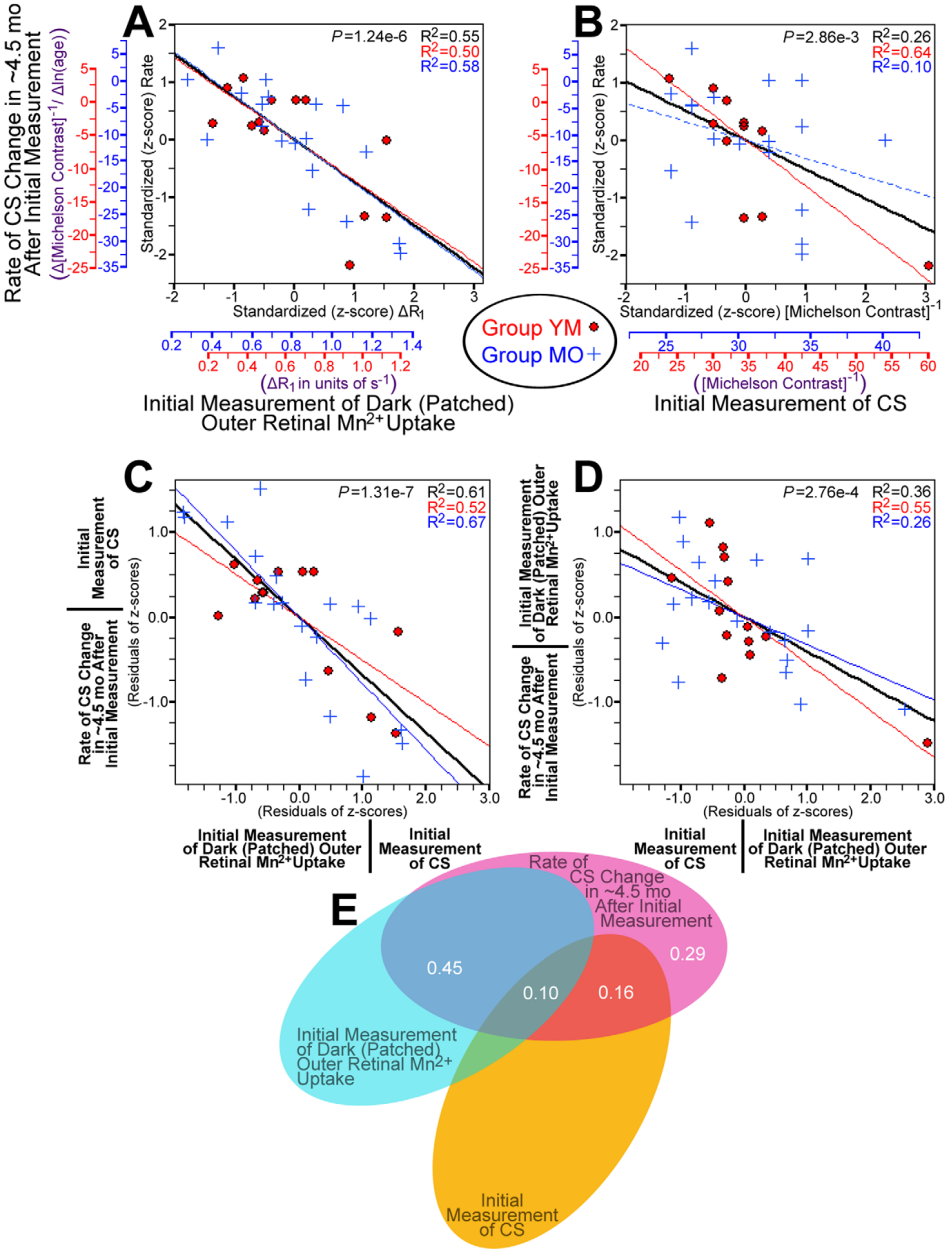
Prediction of CS declines by initial Mn^2+^ uptake and CS. Initial CS and Mn^2+^ uptake measurements were made at age ∼2.5 mo in Group YM (red •) and age ∼7 mo in Group MO (blue +). *A:* Greater Mn^2+^ uptake in the dark-adapted outer retina predicts greater rates of CS decline in the following ∼4.5 mo. The red and blue axes show the pre-standardized values for Mn^2+^ uptake (ΔR_1_; x-axis) and rate of change in CS (change in inverse Michelson contrast divided by change in log-transformed age). *B:* Higher initial CS values predict greater rates of CS decline in the following ∼4.5 mo. *C:* When statistically controlling for initial CS – y-axis values in this plot are the residuals from the combined (standardized; black line) best-fit in *B* – greater Mn^2+^ uptake in the dark-adapted outer retina strongly predicts greater rates of CS declines in the following ∼4.5 mo. *D:* When statistically controlling for the effect of greater Mn^2+^ uptake in the dark-adapted outer retina, initial CS strongly predicts greater rates of CS decline in the following ∼4.5 mo. *E:* Venn diagram showing the proportion of variance in rate of CS decline that is uniquely explained by dark-adapted outer retinal Mn^2+^ uptake (semipartial correlation (‘sr^2^’) = 45%) or initial CS (‘sr^2^’ = 16%), of the total ∼71% of variance explained by some combination of those two variables. The relationship between outer retinal Mn^2+^ uptake and CS declines beyond the ∼4.5 mo period is shown in Supplemental Figure S4 (in File S1). Regression analyses using other MRI measurements (e.g. eye morphology) are provided in Supplemental Tables S1–S4 in File S1.

Having established age-related declines in visual performance together with increases in retinal Mn^2+^ uptake, we next tested whether high Mn^2+^ uptake is linked with age-related declines in visual function. Notably, higher-than-average outer retinal Mn^2+^ uptake on any given rat’s first MRI scan predicted a greater-than-average rate of CS decline *in the ∼4.5 mo following the first MRI scan* (Fig. 4). This relationship was significant when evaluating patched (dark-adapted) eyes, unpatched (light-exposed) eyes, or activity-dependent (dark-light difference) outer retinal Mn^2+^ uptake (all r<−0.50; P<3.1e-3). The initial value of CS was also a significant predictor of CS decline, such that rats beginning the study with higher-than-average CS showed the greater-than-average declines in CS (r = −0.51; P = 2.9e-3; Fig. 4). The strongest predictor of CS declines was Mn^2+^ uptake in the dark-adapted outer retina (*P = *1.2e-6; r = −0.74; Fig. 4), and after statistically controlling for that relationship, only initial CS remained a significant predictor of CS decline (*P = *3.5e-4; Fig. 4). Importantly, the relationship between initial CS and later CS declines (which may be influenced by ‘regression to the mean’) does not account for the relationship between Mn^2+^ uptake and CS declines. After statistically controlling for the relationship with initial CS, each of the Mn^2+^ uptake variables remained a significant (*P*<1.4e-3) predictor of CS decline, with dark-adapted outer retinal Mn^2+^ uptake being the strongest remaining predictor (*P = *2.1e-7; Fig. 4). We note that regression analyses that used both light-adapted Mn^2+^ uptake and the dark-light difference as predictors showed that each was a significant and unique predictor of CS declines, either when statistically controlling for the relationship with initial CS (both *P*<1.8e-4) or not (both *P*<6.7e-4). In short, both initial Mn^2+^ uptake and initial CS are strong and unique predictors of future CS declines in the ∼4.5 mo following the first MRI measurement (Fig. 4).

In Group MO, the third measurement of visual performance revealed an additional pattern: From ∼4.5 to ∼12 mo after the first MRI, rats that had already experienced substantial CS declines (from 0 to ∼4.5 mo post-MRI) tended have their remaining function preserved. However, rats with little-to-no CS declines in the first ∼4.5 mo post-MRI experienced substantial declines thereafter. For this reason, high Mn^2+^ uptake – a strong predictor of CS declines in the ∼4.5 mo following the first MRI – was also a significant predictor of *preserved* CS in the later period (∼4.5 mo to ∼12 mo post-MRI) (see Supplemental Material (Fig. S4, Table S4) in File S1). Taken together, the initial degree of retinal Mn^2+^ uptake strongly predicted the timing of an animal’s transition to its old-adult level of visual performance (i.e., an immediate vs. delayed decline).

### Outer Retinal Mn^2+^ Uptake is L-VGCC-Dependent

**Figure 5:**
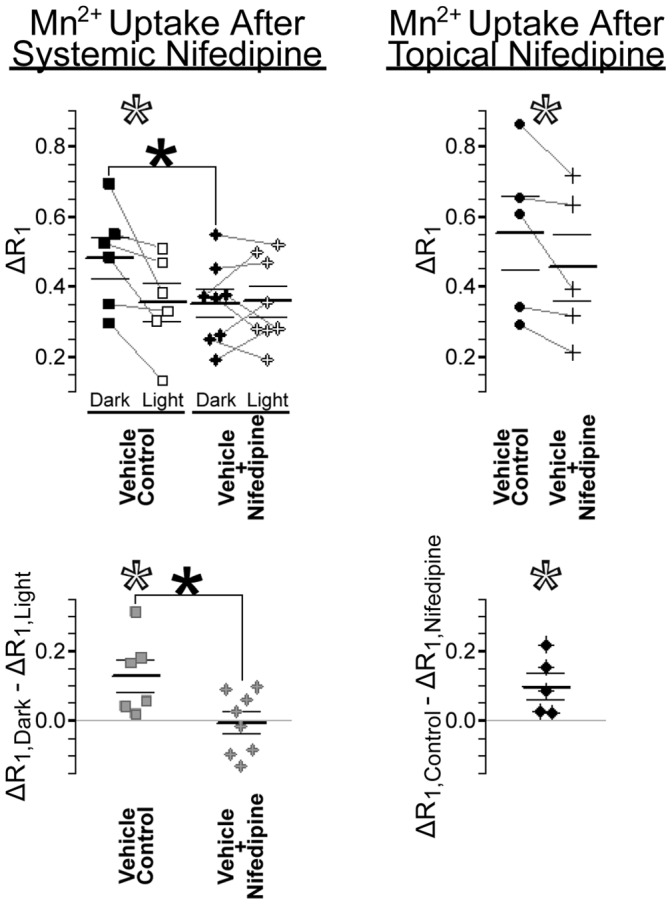
Effects of nifedipine on outer retinal Mn^2+^ uptake. Scatter plots show outer retinal Mn^2+^ uptake (ΔR_1_; in s^−1^) in nifedipine-treated and vehicle control eyes. Inner retinal data are provided in Supplemental Figure S5 (in File S1). Paired measurements (left and right eye of each rat) are connected by grey lines in the top panels, and subtracted from one-another to produce the bottom panels. *Left:* In vehicle controls, dark-light differences are significantly greater than zero (open *s). Systemic treatment with nifedipine significantly inhibited Mn^2+^ uptake, but only in dark-adapted (patched) eyes (top filled *) – thereby significantly reducing dark-light differences (bottom filled *) to 0. *Right:* Mn^2+^ uptake in the dark-adapted outer retina was significantly lower in nifedipine-treated eyes than in the partner vehicle-control eyes (open *s).

Having established a robust link between the extent of retinal Mn^2+^ uptake and future visual declines, we next examined the role of L-VGCCs in photoreceptor (i.e., outer retinal) Mn^2+^ uptake using the specific L-VGCC blocker nifedipine. Consistent with expectations, nifedipine inhibited outer retinal Mn^2+^ uptake in dark-adapted (patched) eyes (*P = *0.042; Fig. 5), but not in light-exposed eyes (*P*>0.5). Further analysis revealed that outer retinal dark-light differences were absent in nifedipine-treated rats (*P*>0.5) but present in vehicle controls (*P = *0.018; Fig. 5), such that ratio (ΔR_1_,_dark_/ΔR_1_,_light_) and difference (ΔR_1_,_dark_–ΔR_1_,_light_) scores differed significantly between the nifedipine and vehicle groups (*P = *0.043 and 0.026, respectively). Analysis with a mixed ANOVA yielded the same result (light vs. dark × drug vs. vehicle interaction: F[_1, 12_] = 6.5; *P = *0.026). In a separate group of rats, we verified that Mn^2+^ uptake was inhibited by local action of nifedipine, rather than possible systemic effects: In dark-adapted rats, nifedipine eye drops significantly inhibited outer retinal Mn^2+^ uptake, relative to measurements from the contralateral vehicle-control eyes (paired t-test: *P = *0.031; Fig. 5). These data confirm a dominant role of L-VGCCs in outer retinal Mn^2+^ uptake.

### Retinal L-VGCC Expression Changes with Age

**Figure 6:**
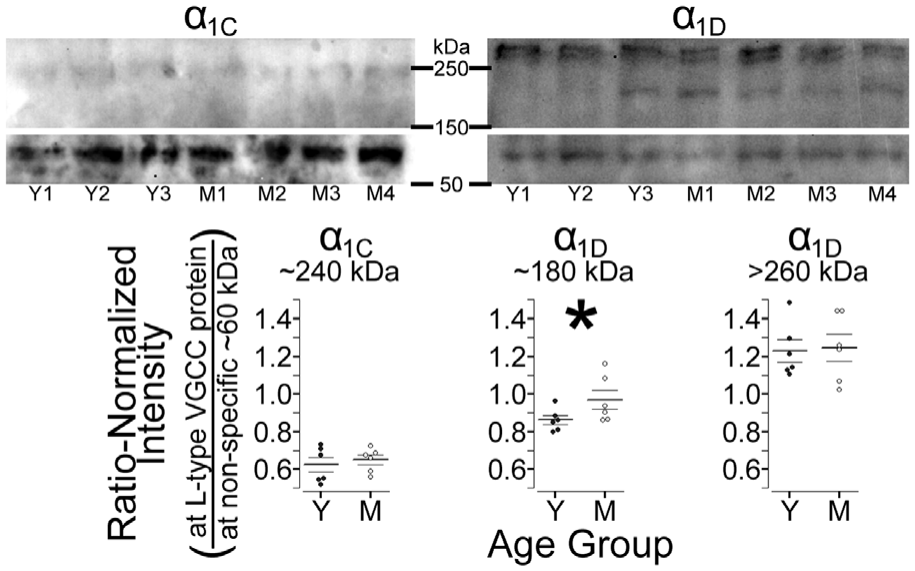
Western blots of retinal L-VGCC expression in young and mid-adult rats. *Top*: Representative anti-α_1C_ (left) and anti-α_1D_ (right) gels with lanes loaded with protein from 3 (of 6) young-adults (Y1–Y3) and 4 (of 6) mid-adults (M1–M4). For display purposes, images were cropped to highlight bands from of L-type channels at >150 kDa and the non-specific band used for normalization at ∼60 kDa. The brightness and contrast settings differ for anti-α_1C_ (meant to display the band at ∼240 kDa) versus anti-α_1D_, but are the same for the high and low molecular weight portions of each gel. *Bottom:* Consistent with expectations, only expression of the ∼180 kDa α_1D_ isoform is higher in mid- than young adults (*P<0.05).

Our longitudinal studies demonstrated an age-related increase in retinal Mn^2+^ uptake, and our experiments with nifedipine demonstrated that a substantial fraction of retinal Mn^2+^ uptake occurs through L-VGCCs. We therefore tested for an age-related increase in L-VGCC expression. Comparing Western blots from young versus mid-adult rat retinas, we found an age-related increase in expression of the ∼180 kDa α_1D_ isoform (*P = *0.043; Fig. 6). Neither the larger (>200 kDa) α_1D_ isoform nor the α_1C_ isoform showed an age-related change in expression (respectively *P*>0.4 and *P*>0.2) – similar to the previously-documented pattern at CA1 of the rat hippocampus [18], [19].

**Figure 7:**
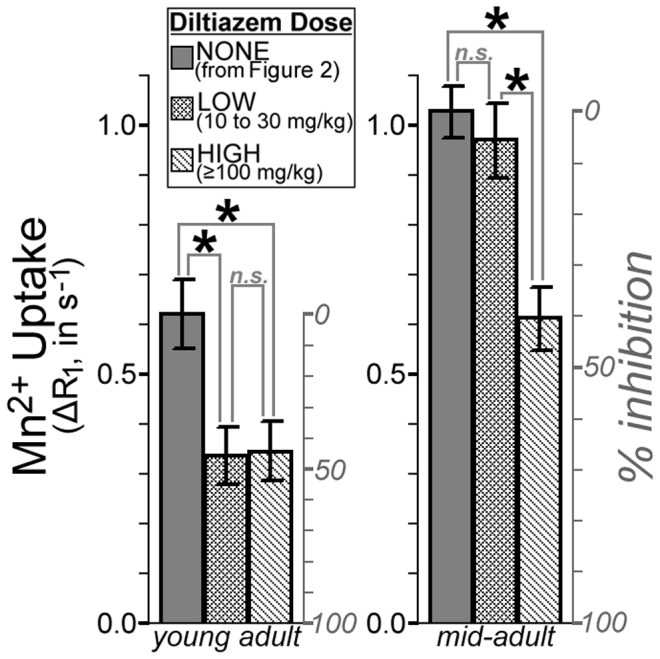
An age-related change in sensitivity to diltiazem, measured *in vivo* with MEMRI. Mn^2+^ uptake in these diltiazem-injected dark-adapted rats is compared to the age-matched dark-adapted control data from our longitudinal studies (shown in Fig. 3). The right y-axis for each plot shows % inhibition, calculated as [1– (ΔR_1,diltiazem/_ΔR_1,control_)]. In young adults, diltiazem significantly inhibits Mn^2+^ uptake (*P<0.05) at both low and high doses: Beyond the ∼40% inhibition seen with the low dose, no additional inhibition is seen with high doses (‘n.s.’ indicates not significant; *P*>0.05). In mid-adults, low doses of diltiazem showed no effect on Mn^2+^ uptake, but high doses yielded significant (*P<0.05; ∼40%) inhibition of Mn^2+^ uptake. Taken together, these data suggest that there is an age-related decrease in sensitivity to L-VGCC blockers, though with high (and not clinically relevant) doses, maximum inhibition is still possible. This pattern is consistent with an age-related increase in the relatively drug-insensitive α_1D_ L-VGCC isoform. Similar results were obtained from the inner retina, and using drug dose as a continuous rather than categorical variable, as shown in Supplemental Figure S6 (in File S1).

We also tested for this age-related, isoform-specific increase in L-VGCC expression *in vivo*: Because the α_1D_ isoform is roughly an order of magnitude less-sensitive to blockade than α_1C_ [20]–[22], an age-related increase in retinal α_1D_ expression should make it more difficult to block Mn^2+^ uptake with an L-VGCC antagonist. We measured retinal Mn^2+^ uptake after injecting low (10–30 mg/kg) or high (100–125 mg/kg) doses of D-*cis*-diltiazem into dark-adapted young and mid-adult rats. In young adults, both low (10–30 mg/kg) and high (100–125 mg/kg) doses of diltiazem inhibited retinal Mn^2+^ uptake by ∼40%, relative to age-matched controls (*P = *5.5e-3 and 4.7e-3, respectively; Fig. 7). In contrast, the low dose of diltiazem had no effect on retinal Mn^2+^ uptake in mid-adult rats (*P*>0.4), but significant inhibition of Mn^2+^ uptake (*P = *1.1e-4) was observed with high doses of diltiazem (Fig. 7). In short, the mid-adult rat retinas appeared less-sensitive to blockade, consistent with Western blot findings of an age-related increase in expression of a drug-insensitive L-VGCC isoform.

## Discussion

Previous studies of the rat hippocampus formed the foundation of the ‘calcium hypothesis of aging’ by demonstrating progressive age-related increases in neuronal Ca^2+^ influx through L-VGCCs, L-VGCC density, and L-VGCC protein expression, which are greatest in those rats with the poorest cognitive function [1]–[5], [18], [19]. In this study, we examined an analogous hypothesis in the neuroretina, and found that age-related declines in CS coincide with increases in retinal ion influx via L-VGCCs. Importantly, the extent of retinal ion influx strongly predicted subsequent rates of CS decline. Furthermore, retinal Mn^2+^ uptake was regulated by age-related and isoform-specific increases in expression of L-VGCCs, which are widespread in the retina [25], [26] and the largest single pathway for neuroretinal Ca^2+^ influx [27]–[29]. L-VGCCs are critical to retinal function [30], [31], and acute L-VGCC blockade improves CS in humans (though the potential influence of age has not been investigated) [32], [33]. Long-term changes in retinal L-VGCCs may also alter growth and remodeling of photoreceptor axons [25]. Together, our data unambiguously extend, for the first time, the ‘calcium hypothesis of aging’ to the neuroretina.

We evaluated neuroretinal ion influx through L-VGCCs *in vivo* with MEMRI, and found the expected pattern of activity-dependent retinal ion influx at all ages (Fig. 3; [16], [34], [35] – low in photoreceptors exposed to light, when membranes are hyperpolarized and L-VGCCs closed, but high in darkness, when photoreceptors are fully depolarized and L-VGCCs open. Mn^2+^ uptake is robustly sensitive to targeted manipulation of L-VGCCs, as demonstrated by reduced retinal Mn^2+^ uptake following L-VGCC blockade with nifedipine (Fig. 5) and diltiazem (Fig. 7). That finding is likely due to local drug effects at the retina, rather than systemic (e.g., cardiovascular [36] effects of L-VGCC blockade, since topical and systemic application of nifedipine yielded similar reductions in outer retinal Mn^2+^ uptake (Fig. 5). We analyzed the role of L-VGCCs on activity-dependent Mn^2+^ uptake, and found that L-VGCC blockade reduced Mn^2+^ uptake in the dark-adapted outer retina to light-adapted levels (Fig. 5). We found that the absolute amount of activity-dependent Mn^2+^ uptake increased with age (Figs. 2, 3). These MEMRI data are in apparent contradiction to results from previous ERG studies in which photoreceptor responses to light *decrease* with age in humans, mice, and pigmented rats [8], [9], [24]). It seems likely that MEMRI and ERG evaluate different aspects of outer retinal ion channel activity: ERG reports on light-dependent changes in photoreceptor membrane voltage, which are driven by Na^+^ (and other cation) entry through cyclic guanosine monophosphate-gated channels [37] while MEMRI analyzes photoreceptors’ dark-light differences in ion influx through Ca^2+^ channels, particularly L-VGCCs. Because ERG declines do not seem to explain visual declines, our present results strongly support MEMRI as a powerful tool for studies into the role of L-VGCCs in aging.

In the rat hippocampus (CA1), expression of L-VGCCs increases with age [1], [18], [19]. To test for this possibility in the retina, we compared Western blots from young and mid-adult adult retinas and found an isoform-specific increase that was strikingly similar to that reported for the rat hippocampus [18], [19]: Expression of the ∼180 kDa α_1D_ isoform increased with age, while expression of α_1C_ and the larger (>200 kDa) α_1D_ isoform appeared independent of age (Fig. 6). Because the α_1D_ isoform is roughly an order of magnitude less-sensitive to pharmacological blockade than α_1C_ [20]–[22], we reasoned that an age-related increase in α_1D_ expression would be revealed by an age-related decrease in drug sensitivity. *In vivo*, we found substantial inhibition of retinal Mn^2+^ uptake with 10–30 mg/kg doses of the L-VGCC blocker diltiazem – consistent with previous work [15] – but only in young adult rats. In mid-adult rats, ≥100 mg/kg doses were needed to produce similar levels of inhibition (Fig. 7). Our data strongly suggest that L-VGCC-based therapies/interventions may become progressively less-effective with age because existing drugs are relatively ineffective at the α_1D_ isoform. The sensitivity of MEMRI to age-related changes in L-VGCCs highlights a potential application of this method for *in vivo* evaluation and optimization of drug efficacy, for instance, complementing ongoing development of L-VGCC blockers specific for the α_1D_-isoform [38]. Although clinically-available L-VGCC blockers seem to have negligible influence on optical factors like lens accommodation (i.e., ciliary muscle contractility [39] and pupil size [40], [41]), and indeed appear to acutely improve vision in humans [32], [33], preclinical evaluation of new α_1D_-selective drugs will likely require assessment of the acute and chronic effects on vision. OKT is a promising approach to aid in such assessments, given our present demonstration that OKT is sensitive to age-related and L-VGCC-associated vision declines.

We now consider the following potential confounders to the interpretation of the MEMRI data: (1) Very high doses of Mn^2+^ carry some risk of toxicity [42], which could negatively impact longitudinal studies. Several lines of evidence argue against Mn^2+^ toxicity in the present study: We have previously demonstrated that the present dose (222 µmol Mn^2+^/kg) is non-toxic to the rat retina, based on measurements of intraocular pressure, blood-retinal barrier integrity, retinal histology [34], electroretinography, and dose-redose reproducibility of retinal Mn^2+^ enhancement [43]. Also, if Mn^2+^ exposure affected long-term visual function, it would be evident in comparisons of ∼7 mo data from Groups YM and MO: At that time point, the former but not the latter had previously been injected with Mn^2+^. *Post-hoc*, we find that Group YM and Group MO have similar visual function at ∼7 mo (for SFT, *P = *0.28; for CS, *P = *0.091; two-tailed t-tests), further arguing against toxicity. Thus, available data overwhelmingly demonstrate that our present dose of 222 µmol Mn^2+^/kg is non-toxic to the rat retina. This is consistent with previous work related to the rat hippocampus, which showed no histological signs of toxicity following a 500 µmol Mn^2+^/kg injection [44], and normal performance on a memory task after a 200 µmol Mn^2+^/kg injection [45]. (2) Longitudinally, we found that Mn^2+^ uptake increased with age. If Mn^2+^ efflux rates were slow enough, then the second measurement might be higher than the first merely because residual Mn^2+^ remains from the first injection. This seems unlikely, given that the half-life for retinal Mn^2+^ efflux is less than one day [16]. Furthermore, the longitudinal finding of higher Mn^2+^ uptake in mid- than young-adult rats (Group YM) can be reexamined by cross-sectional comparison of the once-injected ∼2.5 mo rats from Group YM to the once-injected ∼7 mo rats from Group MO: Those *post-hoc* comparisons yield significant differences for both inner and outer retina in both dark and light (all *P*<1.5e-3), consistent with the longitudinal findings (Fig. 3). (3) Finally, any declines in blood-retinal barrier (BRB) integrity might alter neuronal Mn^2+^ exposure, and therefore uptake. We tested for vitreous enhancement following injection of the intravascular contrast agent Gd-DTPA, and found no signs of BRB compromise during healthy aging (see Supplemental Figure S2 in File S1), consistent with previous work [46].

The present results may explain why prior studies of the anatomy and physiology of the aging retina have had little success explaining age-related declines in visual function (see [10] for review). For example, as detailed in the Supplemental Material (Fig. S3 in File S1), *in vivo* measurements of retinal morphology did not indicate volume loss with age, suggesting that anatomical measurements aimed at detecting neuron loss would give little insight into vision declines in healthy aging. Also, previous *in vivo* physiologic measurements using ERG are likely insensitive to the important changes in L-VGCCs found herein, which strongly predicted the rate of age-related declines in CS (Fig. 4). Our demonstration that the ‘calcium hypothesis of aging’ applies to the retina offers a new approach for understanding age-related declines in visual function – one which might be pursued in translational clinical studies using the FDA-approved Mn^2+^-based contrast agent Teslascan [16].

## Materials and Methods

Male Long-Evans rats (Hilltop Labs; Scottdale, PA) were studied. Rats were given food and water *ad libitum* and housed and maintained in normal 12 h light/12 h dark cycling. Ages, weights, etc. in this section are reported as mean(standard deviation). All animals were treated in accordance with the National Institutes of Health Guide for the Care and Use of Laboratory Animals, the Association for Research in Vision and Ophthalmology Statement for the Use of Animals in Ophthalmic and Vision Research, and authorization by the Institutional Animal and Care Use Committee (IACUC) of Wayne State University.

### Animals Studied Longitudinally

Group YM was studied from *Y*oung to *M*id adulthood, and Group MO was studied from *M*id to *O*ld adulthood. At the first and final time points for each group, tests of visual performance were followed (∼1 wk later) by high-resolution MEMRI measurements of both eyes (details below).

The need for high-quality longitudinal MRI data from both eyes of each rat resulted in some missing or excluded data, due, for instance, to anesthetic-related death. When possible, outcome metrics between rats lost to follow-up (missing/excluded) and rats retained for the duration of the study were compared, and as suggested by visual inspection of scatter plots (Figs. 1, 3, and Fig. S3 in File S1), no statistically significant differences were noted.

We started with a total of 42 rats in Group MO. Three rats were excluded from the final analysis because they had unusually and unilaterally thick retinas (|left-right thickness difference| of (mean(SD)) 72(7) µm, versus 9(8) µm in the other rats) to the degree that they were univariate outliers (|z|>3.3). This asymmetry was not related to the eye patch or duration of anesthesia: The thicker retina was in the unpatched eye in one of the three cases, and in the first eye scanned in two of three cases. The remaining 39 rats in Group MO, were 206(10) d old, and weighed 439(50) g at the first time point. In 32 of those rats, high-quality MRI data was collected from both eyes at the first time point. Roughly half were available at the intermediate time point, 144(13) d later, when only visual performance and body weight were measured (n = 19; age 350(15) d; 580(50) g). Fifteen of those rats contributed data to the final (old adult) time point (age 580(29) d; 605(67) g). Of those fifteen, high-quality MRI data could not be collected for one, and another three were randomly selected to be scanned *without* Mn^2+^ injection, so as to measure baseline retinal R_1_ in old adulthood. In summary, paired (longitudinal; mid- vs. old adult) comparisons of visual performance were performed in 15 Group MO rats, 14 of which were used for comparisons of eye morphology, and 11 for comparisons of retinal Mn^2+^ uptake.

We started with 22 rats in Group YM. One rat had unilaterally thickened retina and was excluded from all analyses. Of the remaining 21 rats (initial age of 73(9) d; weighing 290(70) g), 18 provided high-quality MRI data at the first time point. Thirteen of those were available for follow-up in mid-adulthood (aged 227(7) d; 502(34) g), of which 11 provided high-quality MRI data. In summary, paired (longitudinal; young vs. mid-adult) comparisons of visual performance were performed in 13 Group YM rats, 11 of which were used for comparisons of both eye morphology and retinal Mn^2+^ uptake.

### Visual Performance

Visual performance was measured in awake and free-moving animals using OKT, which utilizes rodents’ reflexive head movements in the presence of a moving sine wave grating. Head movements are present when stimuli are clearly visible, but absent when the experimenter makes stimuli too difficult to see, either due to low contrast when measuring CS, or high spatial frequency when measuring SFT. Further details on OKT, including video-recorded examples of the characteristic head movements and experiments on the neuronal pathways involved in OKT responses, are available through the work of Douglas, Prusky, and colleagues (e.g. [47], [48]). In the present studies, SFT and CS were measured on an in-house built OKT device (Fig. 1, File S1 in File S1). Rats had unrestricted head movement during each of the eight 15–20 min sessions (1–2 per day) used to measure SFT (4 of the 8 sessions) and CS at a given age. The best performance recorded for an animal during these sessions (confirmed by multiple tracking events at the same OKT device setting) was used for statistical comparisons.

### Eye Patch

An opaque eye patch was used to keep one eye dark-adapted while the other was exposed to normal lab lighting (∼300 l×) during Mn^2+^ uptake. The eye to be patched was alternated from rat to rat, so that roughly half the members of each group had the left eye patched at each time point. The day before MRI scans, the patch was adhered to one side of the head while the rat was anesthetized with diethyl ether, as previously described [35]. Briefly, after the selected eye was gently sutured shut and protected by application of puralube (Pharmaderm; Melville, New York), the patch was adhered with a combination of eyelash glue (Andrea modlash adhesive; American International Industries, Los Angeles, CA) and spirit gum (Mehron Inc., Chestnut Ridge, NY). Each rat was fit with an Elizabethan collar and monitored until recovery from anesthesia (≤15 min). Rats were then dark-adapted overnight with free access to food and water. In all cases, sutures remained intact and patches remained fully-adhered until MRI scans on the following day.

### Acquisition of MEMRI Data

Prior to i.p. injection of 44 mg MnCl_2_·4H_2_O/kg body weight (0.1 M solution in 0.9% saline), rats were brought into normal lab lighting for 30 min. After Mn^2+^ injection, rats continued their monocular exposure to normal lab lighting for ∼4 h – our standard post-injection time span, selected to ensure full retinal Mn^2+^ uptake while animals remained awake and freely moving [14]–[16], [34]. MRI readout of retinal Mn^2+^ uptake was performed under dim red light or darkness, and began immediately after that ∼4 h period, when rats were anesthetized with a ketamine/xylazine (‘k/x’) solution. Maintenance doses of k/x were administered *in situ* as needed through an intraperitoneal line accessed from just outside the magnet bore.

MRI scans of the left eye began 4.4(0.4) h after Mn^2+^ injection, and were immediately followed by scans of the right eye (5.5(0.7) h post-Mn^2+^). For each eye, tissue R_1_ ( = 1/T_1_) was measured as follows: Using a 1.0 cm diameter receive-only coil on a 7 T Bruker ClinScan system, retinal images were collected at eight different TRs with a standard spin-echo sequence (echo time (TE) 13, 160×320 matrix, slice thickness 600 µm; 8×8 mm^2^ field of view; yielding an in-plane resolution of 50 µm from superior to inferior×25 µm in the axial (optic nerve to cornea) direction). Multiple repeat images were collected at lower TRs (total number given in brackets), then registered and averaged offline to improve signal-to-noise. Images were collected in the following order: TR 0.15 s [6], 3.50 s [1], 1.00 s [2], 1.90 s [1], 0.35 s [4], 2.70 s [1], 0.25 s [5] 0.50 s [3].

### Acquisition of Baseline (no Mn^2+^) Data

To aid in the interpretation of MEMRI data, the above procedures for measuring R_1_ in a light-exposed and dark-adapted eye of the same animal were performed in 9 rats *without* Mn^2+^ injection. The patched and unpatched eyes of young (n = 3, aged 74(5) d, weighing 296(17) g), mid- (n = 3, 198(7) d, 417(25) g) and old adult rats (those randomly selected from Group MO; n = 3, 570(29) d, 557(60) g) were scanned under k/x anesthesia, starting 5.1(0.6) h after exposure to normal lab lighting began. This timing closely matched that used for Mn^2+^-injected rats. Because baseline R_1_s were stable with age and light exposure (Fig. 2), we averaged across ages and lighting conditions to generate a single baseline outer retinal R_1_ (0.568 s^−1^), which was subtracted from Mn^2+^-enhanced R_1_s to calculate ΔR_1_s. In a subset of these baseline rats (2 young, 1 mid, 2 old), R_1_ measurements were followed by evaluation of blood-retinal barrier (BRB) integrity using dynamic contrast enhanced MRI as detailed in Supplemental Material (Fig. S2 in File S1).

### Spatial Normalization of Retinal Data and Analyses of Morphology

Previous rat (and human) studies have demonstrated decreases in retinal thickness and increases in retinal surface area throughout adulthood (e.g., [49]–[52]). Spatial normalization of retinal data is therefore an important precursor to physiological (i.e., Mn^2+^ uptake) comparisons, and was performed as detailed by Bissig & Berkowitz [35] using semi-automated R (http://www.r-project.org) scripts developed in-house. Briefly, polynomials were fit to the to the vitreoretinal border, then integrated about the central axis of the eye to calculate retinal surface area and (in combination with retinal thicknesses; see below), retinal volume. In-plane, signal intensities were sampled along perpendiculars to the polynomials, then organized as a linearized image of the retina. The distance from optic nerve to ciliary body was measured for each hemiretina, and values were averaged to calculate retinal extent. The linearized retina was then binned in 10% increments of that distance, % extent, with 0% extent at the optic nerve head, and 100% extent at the ora serrata. Average signal intensity as a function of retinal depth was calculated for each % extent bin, producing a signal intensity profile. Vitreoretinal and retina/choroid borders were demarked in each profile (where signal intensity fell halfway between the local minimum and maximum) and subtracted to calculate retinal thickness. Profiles were then resampled from a µm scale to a % thick scale, with 0% thick at the vitreoretinal border, and 100% thick at the retina/choroid border, in 4% thick increments. These spatially-normalized signal intensity profiles facilitated comparisons of retinal Mn^2+^ uptake: Although the retina thins with age and distance from the optic nerve, the relative (% thick) position of each retinal layer is stable in healthy adults [49], [53]. We report average retinal thicknesses and tissue Mn^2+^ uptake for the central retina (10–30% extent).

Retinal morphology, produced as a byproduct of spatial normalization and not the focus of the present work, is summarized in the Supplemental Material (Fig. S3 in File S1). There, we also provide measurements of eye size, optical components, and the resulting estimates of refractive state [54]. We note that regression analyses revealed no consistent relationships between eye/retinal morphology and retinal Mn^2+^ uptake (Supplemental Tables S1, S3, and S4 in File S1).

### Analysis of Retinal Mn^2+^ Uptake

As described above, central retinal signal intensity (‘SI’) profiles were measured at each TR. These signal intensities were used to calculate tissue T_1_ based on the equation SI = a+b * (exp(-TR/T_1_)), where a, b, and T_1_ are fitted parameters. Data were fit with this function by the Levenberg-Marquardt nonlinear least-squares algorithm using the minpack.lm library for R. Binned central retinal data were then averaged to produce a single profile of T_1_ as a function of depth into the retina (i.e., % thick). T_1_s from 16–28% thick were averaged to represent inner retina, while data from 48–68% thick were averaged to represent outer retina. These spans respectively fall within the inner plexiform and outer nuclear layers, based on *in vivo* OCT images of Long-Evans rat retinas [55]–[57]. The inverse of T_1_ is R_1_, which varies linearly with tissue Mn^2+^ concentration [58]. Mn^2+^ uptake was measured by calculating the difference between Mn^2+^-enhanced R_1_ and the baseline (i.e., without Mn^2+^ injection) retinal R_1_, yielding the measurement ΔR_1_. Based on measurements from the rat brain [58], ΔR_1_s of 0.0, 0.5, and 1.0 s^−1^ represent tissue Mn^2+^ concentrations of 0, 80, and 160 µM respectively.

### Statistics for Longitudinal Studies

We began by evaluating baseline (i.e., no Mn^2+^) R_1_s. Since these data were collected in young, mid-, and old-adult rats, we first tested (linear regression) for effects of age by looking for significant correlations between age and either R_1,Dark_, R_1,Light_, or the dark-light difference (R_1,Dark_–R_1,Light_). These analyses suggested, as expected, no effect of age on baseline values (see legends for Fig. 2 as well as Fig. S3 in File S1). Since we also found no effect of light on baseline R_1_s (two-tailed t-tests; n = 9 pairs of eyes), we averaged across ages and lighting conditions to generate a single baseline value for inner retinal R_1_ (0.514 s^−1^) and outer retinal R_1_ (0.568 s^−1^). These R_1_s were subtracted from the Mn^2+^-enhanced R_1_s, discussed next, to generate ΔR_1_s.

Based on previous *in vivo* MEMRI studies (e.g., [34], [35]) demonstrating that photoreceptor ion influx is greatest in darkness when photoreceptors there are fully depolarized [59], *outer* retinal Mn^2+^ uptake is expected to be higher in the patched, dark-adapted eye than in the unpatched, light-exposed eye. Here, we performed one-tailed paired t-tests to test for that pattern. We also generated ΔR_1,Dark_/ΔR_1,Light_ ratios, and tested whether they differed significantly from 1 (one-sample t-tests).

Next, we tested for longitudinal changes in each variable. Paired two-tailed t-tests were used to compare the first to final time point within each group, testing, for instance, whether retinal Mn^2+^ uptake increased significantly between ages ∼2.5 and ∼7 mo in Group YM. To control for type I error in these multiple comparisons, only results falling below a standard false discovery rate threshold (‘FDR’) were considered significant (q = 0.05 on 52 tests (including comparisons of retinal morphology, etc.; see Fig. S3 in File S1); [60]).

Linear regression was used to test whether the initial value of a given variable predicted the rate of change in a given variable. For instance, do rats with above-average Mn^2+^ uptake in young-adulthood experience above-average declines in CS in the subsequent 4.5 mo? For a given time span and variable (‘VAR’), rates were calculated as (VAR_final_ – VAR_initial_)/(ln(age_final_) – ln(age_initial_)). For Group MO, average rates of change from study start to study end in SFT, CS, and body weight – variables which were also measured at an intermediate time point ∼4.5 mo after the initial MRI – were treated in the same way, but using a linear best fit (after log-transforming age) to the three available measurements.

Additional regression analyses were used, for instance, to test whether two variables are correlated with one-another at the first time point. They are detailed as Supplemental Material (Tables S1–S4 in File S1).

Where possible, regression analyses are performed after combining data from Groups YM and MO: Values from each group at each age – and for rates at each time span – are standardized by conversion to z-scores: For instance, a Group YM subject’s standardized retinal thickness at age ∼2.5 mo is ([subject’s retinal thickness]-[mean of YM retinal thicknesses at ∼2.5 mo])/[SD of YM retinal thicknesses at ∼2.5 mo]. Standardizing scores has no effect on p-values or correlation coefficients (Pearson’s r) when testing one group at a time, but allows both groups to be combined in the same analysis without biasing the outcome due to differences in group means or variances. Before finalizing comparisons, we also tested for Group × variable interactions. These could occur if, for instance, Mn^2+^ uptake was related to CS in mid-, but not young, adults. In the presence of a suspected interaction (*P*<0.05; not corrected for multiple comparisons) groups were analyzed separately. When there was no evidence of an interaction (*P*>0.05) formal statistical testing used only the combined (YM and MO) analysis. For completeness, though, we report correlation coefficients from each group.

Regression results were considered significant below a standard FDR threshold (q = 0.05; see Tables S1–S4 in File S1 for total number of tests). Some *post-hoc* testing with multiple regression was used to further interpret positive results, and exact p-values are reported in those cases.

### MRI Experiments with L-VGCC Blockers

Unless otherwise noted, all aspects of these experiments – for instance, the eye patch procedure, Mn^2+^ doses, MRI procedures and image processing, including the use of the averaged baseline (no Mn^2+^) data described above to calculate ΔR_1_s – are identical to those used in our longitudinal studies. L-VGCC blockers were purchased from Sigma-Aldrich (St. Louis, MO).

#### Experiment with i.p. nifedipine

Retinal Mn^2+^ uptake was measured in both light- and dark-adapted eyes using two groups of young-adult rats. After a patch was applied to one eye of each rat (for four members of each group, the right eye), animals were dark-adapted overnight. Immediately after beginning monocular light (∼300 l×) exposure on the following day, drug-treated rats (n = 8; 264(20) g; aged 63(3) d) were injected with nifedipine (i.p., 30 mg/kg) dissolved in DMSO (20 mg nifedipine/ml of undiluted dimethyl sulfoxide), and vehicle-control rats (n = 6; 259(57) g; aged 63(9) d) were injected with DMSO only (1.5 ml/kg). Each rat was injected with Mn^2+^31(2) min later, then maintained in normal lab lighting until the start of anesthesia (k/x) and immediate MRI scanning of the left, then right, eyes (respectively 4.3(0.2) and 5.5(0.2) h after Mn^2+^ injection in drug-injected and 4.4(0.3) and 5.6(0.3) h in controls).

#### Experiment with topical nifedipine

The influence of nifedipine eye drops on retinal Mn^2+^ influx was tested in five dark-adapted young adults (330(18) g; aged 80(2) d). Undiluted PEG400 was used as a vehicle for the extremely hydrophobic nifedipine. All procedures took place under dim red light or darkness. After anesthetizing the rat with diethyl ether, six 50 µl drops of nifedipine solution (0.211 M) were applied to one eye (the right in 3 of 5 subjects; ∼1 min between each drop), and six 50 µl drops of vehicle (PEG400 only) were applied to the contralateral eye – allowing for paired comparison of nifedipine-exposed to control eyes. No signs of irritation were noted when following application of PEG400 and nifedipine, consistent with previous work [40], [61]. Rats were injected with Mn^2+^38(2) minutes after nifedipine exposure (ensuring full recovery from anesthesia), then scanned ∼4 h later (left and right eye respectively at 4.0(0.3) and 5.3(0.5) h post-Mn^2+^). Scans began immediately after inducing urethane anesthesia (3.7(0.3) ml/kg of a 36% w/v solution in 0.9% saline). Only dark-adapted retinas were studied in this experiment; eye patches were not applied to these rats.

#### Experiment with i.p. diltiazem

We used D-*cis*-diltiazem (i.e. the (+)-*cis* isomer, which has high affinity and specificity for L-VGCCs [62], [63] for this dose-response experiment based on our prior experience with this drug in young adult rats [15]. Young adult (n = 7, aged 72(2) d, weighing 287(26) g) and mid-adult (n = 10, 202(20) d, 465(59) g) rats were dark-adapted overnight. The following day, they were injected with 10, 30, 100, or 125 mg diltiazem (in 0.9% saline; 1.4 ml/kg body weight), and maintained in darkness until MRI scanning was complete. Rats were injected with Mn^2+^31(2) min after diltiazem injection. 4.0(0.3) h after Mn^2+^ injection, the left eye of these dark-adapted rats was scanned under urethane anesthesia (see above; 3.6(0.8) ml/kg). We used age-matched dark-adapted (patched) data from our longitudinal studies as a no-drug control: Young-adult control data was provided by the first time point in Group YM, while mid-adult control data came from the second time point in Group YM and first time point in Group MO.

#### Statistics

Multiple *in vitro* [11], [12] and *in vivo* [13], [15] studies have demonstrated that L-VGCC blockade inhibits Mn^2+^ uptake. In addition, several previous studies, both *in vivo* with MEMRI [16], [34], [35] and *ex vivo* (see [59] for review), have demonstrated that the outer retina is more ion-permeable in dark than light. For these experiments with L-VGCC blockers, it was therefore appropriate to analyze Mn^2+^ uptake (ΔR_1_) with one-tailed t-tests (patched>unpatched; vehicle-control>nifedipine-exposed; control>low-dose diltiazem>high-dose diltiazem; α = 0.05). The raw data (scatter-plots) and exact p-values are provided for these tests. We emphasize that the effect of L-VGCC blockade on Mn^2+^ uptake was highly reproducible across drugs and delivery methods, and the dark-light difference found in controls (for the i.p. nifedipine group) matched findings from our longitudinal studies. Other statistical tests, used sparingly to aid in the interpretation of results, are detailed in Results or Supplemental Material sections.

### Western Blots

Retinal expression of two isoforms of the pore-forming subunit of L-VGCCs – α_1C_ and α_1D_ (i.e.Ca_v_1.2 and Ca_v_1.3, respectively) – was measured in young adult (n = 6; all age 59 d, weighing 267(9) g) and mid-adult rats (n = 6; 192 d; 532(53) g). Immediately after death via urethane overdose, the left retina was isolated from each rat and stored at −80°C. Later, samples were sonicated on ice in a nonionic denaturing urea buffer (6 M urea; 62.5 mM Tris-HCl; pH 6.8; 10% glycerol; 2% sodium dodecyl sulfate; 0.00125% bromophenol blue; and freshly-added 5% β-mercaptoethanol; [64], assayed for protein concentration [65] and diluted with the urea buffer to 3 µg protein/µl in preparation for SDS-PAGE. Samples (60 µg protein per lane) and size standards (Bio-Rad Dual Color Standards #161-0374) were loaded onto 6% polyacrylamide gels, then separated and electrotransfered onto PVDF membrane (Immobilon-P, Millipore Corp., Bedford MA). Membranes were blocked at room temperature in TST (10 mM Tris base and 145 mM NaCl in dH2O, pH 8.0, mixed with Tween 20 (0.05% v/v)) containing 10% w/v non-fat dry milk and 3% w/v BSA, then incubated overnight at 4°C with primary antibodies – mouse anti-β actin (clone AC-15, #A5441, lot 030M4788, 1∶10,000 dilution, Sigma-Aldrich) and either mouse anti-α1C (clone L57/46, #73-053, lot 437-4VA-62, 1∶4 dilution) or mouse anti-α1D (clone N38/8, #73-080, lot 437-4VA-10, 1∶4 dilution) obtained from the UC Davis/NIH NeuroMab Facility – mixed into TST containing 5% w/v nonfat dry milk and 1.5% w/v BSA. The following day, blots were thoroughly washed in TST, then incubated for 1.5 h at room temperature with horseradish peroxidase-linked sheep-anti-mouse IgG (#NA931V, lot 399402, 1∶5000 dilution, GE Healthcare), mixed into TST containing 5% w/v non-fat dry milk and 1.5% w/v BSA. Blots were visualized using chemiluminescent horseradish peroxidase substrate (#WBKLS0500, Millipore) and digitally captured using Fluor-Chem E camera system (Protein-Simple Co., Santa Clara CA).

Blot chemiluminescence intensities were quantified in ImageJ. Although we initially considered using β-actin for normalization, this was not practicable due to overly intense β-actin signal [66], which we attribute to the relatively large sample loadings needed to quantify expression of the less-abundant L-VGCCs. Intensities were therefore normalized to a non-specific band common to both anti-α_1C_- and anti-α_1D_-exposed blots at ∼60 kDa, as in [67].

#### Statistical analysis of western blots

The direction of findings for the ∼180 kDa isofrom of α_1D_ was predicted both by two previous studies in CA1 of the rat hippocampus [18], [19], and significant differences in diltiazem dose-response data (see Results). We therefore used one-tailed t-tests (mid>young adults, α = 0.05) to test for age differences in expression of that protein. The other bands (α_1C_, and α_1D_ banding at >200 kDa) were similarly tested, but served as negative controls: Only the ∼180 kDa α_1D_ isofrom was expected to change with age [18], [19].

## Supporting Information

File S1 This file contains the following figures: **Figure S1, related to** **Figure 1:** **Details of the in-house-built OKT device.** The device was made of three identical 19′′ LCD monitors arranged in an equilateral triangle. Mirrors placed on the floor and ceiling reflected a moving sine wave grating, displayed and distorted identically on each screen with Vision Egg (v.1.0), thereby forming a virtual barrel (i.e., the width of one dark-light-dark cycle appearing similar in all directions) when viewed from the center of the device. A: An overhead view with screen faces represented by solid black lines, and screen edges connected by dashed lines (the corners of the device are occupied by screen casings, which are not shown in this panel). The rat’s right eye is shown just inside of the bulging-triangle shape that defines the testing arena. Tracking was recorded only if the rat remained on the perch and the stimulated eye (left eye if the virtual barrel was moving clockwise; right if counter-clockwise) was inside the arena. The arena was marked only on the operator’s screen, superimposed on and calibrated to the overhead video feed (Microsoft LifeCam VX-2000). Azimuth – direction in the horizontal plane with 0° at the center of the lower left screen – is used as the x-axis for panel C. B: Side view, with one screen removed. The perch was constructed of a single piece of thick metal wire, padded at the hind- and forelimb placements, and secured at the center and one corner of the device floor. C: As a rat’s eye moves closer to a screen, less of the nearest dark-light-dark cycle fits within a degree visual angle. Measuring from the position of the rat’s left eye in panel A, and a device setting of 0.111 c/bd (used for all CS measurements), stimuli at 0° azimuth will have the spatial frequency of 0.091 cycles per degree visual angle. This is plotted as a cycles-per-degree-visual-angle to cycles-per-barrel-degree ratio of 0.82( = 0.091/0.111). Near the corners of the device (gaps at −60° and 60° represent screen casings), ratios of ∼1.08 indicate that the 0.111 c/bd stimulus has a subjective spatial frequency of (1.08 = 0.120/0.111) 0.120 cycles per degree visual angle. For the right eye, which is closer to the screen center at 0° azimuth, a 0.111 c/bd setting means that stimuli will subjectively range from ∼0.076 to ∼0.133 cycles per degree visual angle. In this way, a single device setting can cover the range of frequencies in which Long-Evans rats’ contrast sensitivity is best (Douglas et al., 2005). D: (Left): Reproducibility of SFT measurements on our in-house device was assessed in 13 mid-adult Long-Evans rats (mean(SD) age of 206(14) d, weighing 458(67) g) that were not used in any other experiments. Consistent with expectations – and demonstrating good reproducibility – we found a significant (P<0.05) correlation between the first SFT measurement (the maximum of 4 sessions) and the second SFT measurement 14(10) d later, with no net change in SFT (P = 0.296; paired two-tailed t-test) in the short time between test and retest. D: (Right): Late in the present studies, a commercial OKT system (OptoMotry; CerebralMechanics, Lethbride, Alberta, Canada) became available. On that system, the stimuli are dynamically altered based on eye position, such that the rat always sees 0.5 cycles/degree visual angle when the machine is set to 0.5 c/bd. As noted with panel C, no such dynamic adjustments were possible on the in-house system, thereby allowing some dark-light-dark cycles to subjectively appear broader (easier to see when testing SFT), depending on eye position. For this reason, the threshold c/bd values for the in-house system were expected to be higher than for the commercial system. We measured SFT of 9 young adult rats and 13 old adult rats using both the in-house and commercial systems (≤10 d between measurements). Shortly after this vision testing, the young adult rats were used for the diltiazem MEMRI studies detailed in the main text, and old adult rats were used for the final longitudinal time point (Group MO). Consistent with expectations, rats had lower apparent SFTs (in c/bd) on the commercial system than on in-house system. Importantly, SFT measurements were well-correlated (P<0.05) between systems. We note that our SFT measurements on the commercial system (∼0.54 cycles/deg.vis.angle) are in good agreement with those obtained from Long-Evans rats in previous studies [1], [2]. Finally, note that the aging pattern for SFTs described in the main text (young>mid; young>old; mid ≈ old) is well-replicated by the data shown in panel D. **Figure S2, related to** **Figure 2:** **The blood-retinal barrier (BRB) is intact throughout adulthood.** BRB integrity was evaluated in five of the nine rats (2 young, 1 mid-, and 2 old adults) used to collect baseline (no Mn^2+^) measurements of tissue R_1_. BRB measurements were performed at the very end of each rats’ scanning session (i.e. after all R_1_ measurements were complete). As in [3], BRB was measured by testing for vitreous enhancement in T_1_-weighted images following tail vein injection of the vascular contrast agent Gd-DTPA (0.3 mmol/kg; Magnevist; Bayer HealthCare Pharmaceuticals, Leverkusen, Germany): Using the same hardware as for all other MRI scans reported in the main text, 25 images of the eye were collected over a 37 min period using a standard spin-echo sequence but with much lower spatial resolution than the MEMRI studies (TR 1.0 s, TE 11, 64×128 matrix, slice thickness 600 µm; 12×12 mm^2^ field of view; 1 min 18 s acquisition time, 15 s pause between scans).The bolus of Gd-DTPA was injected through a tail vein between the fifth and sixth images. Loss of BRB integrity would be demonstrated by gradual leakage of Gd-DTPA into the vitreous, causing enhancement in these T_1_-weighted images. *Top:* A hand-drawn region of interest (red outline) was used to measure the mean signal intensity of all vitreous (‘vSI’) appearing in each image. On visual inspection of these representative image series from a young and an old adult rat, no vitreous enhancement occurred after Gd-DTPA injection, arguing that the BRB is fully intact. The following areas do not have blood barrier and act as positive internal controls: the aqueous humor (green arrow) and extraocular tissues (blue arrows) clearly enhanced (higher signal intensity) after Gd-DTPA injection. *Bottom:* Time-courses of vitreous signal intensity (‘vSI’) are shown after normalizing to pre-contrast signal intensities. For each subject, we calculated the mean and standard deviation vSIs from the five pre-Gd-DTPA images (i.e., ‘vSI_mean(pre)_’ and ‘vSI_sd(pre)_’). The difference between vSI at a given time (vSI_t_) and vSI_mean(pre)_, when divided by vSI_sd(pre)_ (i.e. (vSI_t_ – vSI_mean(pre)_)/vSI_sd(pre)_) produces a standardized vitreous signal intensity at each time point for each rat. Analogous to a z-score, values between −1.96 and 1.96 are within the 95% prediction interval of pre-Gd-DTPA data. Only post-Gd-DTPA standardized values rising *and consistently remaining* above ∼2 would suggest vitreous enhancement. None of the five subjects showed signs of vitreous enhancement post-Gd-DTPA injection, demonstrating an intact barrier to passive leakage. In an alternative approach (not shown) we calculated passive BRB permeability surface area products (‘BRB PS’; based on Berkowitz et al., 2004) for each rat, but found no relationship between BRB PS and age with linear regression (r = 0.03; *P* = 0.97), and found that all five rats had BRB PS values less than or equal to previously-collected control rat data [3]. **Figure S3, related to** **Figure 3:** **Longitudinal analyses of morphology and inner retinal Mn^2+^ uptake.** *Images:* Good contrast between ocular tissues was obtained on TR 1.0 s T_1_-weighted images facilitating morphology evaluation. A representative eye image is shown here twice, with different brightness/contrast levels to aid in label visibility. An enlarged section of the retina (red box) shows that retinal layering is clearly visible with the present imaging resolution. For each eye, semi-automated scripts written in R (v.2.9.0; http://www.r-project.org) were used to measure anterior chamber depth, lens thickness, posterior chamber depth – the sum of those three measurements being axial length – as well as the radii of curvature (‘rC’) for the anterior lens surface, posterior lens surface, and external surface of the cornea. *Scatter Plots (all):* As in main text Figures 1 and 3, mean±SEM are indicated by the horizontal lines overlaid on each scatter plot. Longitudinal measures from the same subject are connected by gray lines, while a white mark within a point denotes the lack of follow-up data (e.g., due to animal death). Note that values from rats lost to follow-up are evenly distributed among those from animals retained for longitudinal comparisons. Animal age and group membership are noted at the bottom of each scatterplot. Including the data shown here, and the data in main text Figures 1 and 3, we performed 52 paired two-tailed t-tests of longitudinal changes. § indicates a significant (q<0.05; calculated on the 52 tests) effect of age. For body weight, the significance test for ∼7 vs. ∼19 mo data is not labeled since the result (*P = *5.1e-8) is well-depicted by the ∼7 vs. ∼11.5 mo comparison. The interrelationship between these variables was detailed using regression analyses, with results given in Tables S1–S4. *Scatter Plots (Optics):* With the exception of vitreous chamber depth, each measure of eye morphology showed significant increases with age – consistent with continued and well-coordinated eye growth throughout adulthood. Measurements of size and position of optical components also allowed us to calculate refractive state using Hughes’s two shell (“core”) lens model, and the refractive indices reported therein. As in our recent application of that model to MRI images of the juvenile rat eye [4], corneal thickness was estimated by its ratio to axial length (axial length/corneal thickness = 23.004; [5]), and the diameter of the spherical lens core, which is centered in the lens, was set proportional to (based on [5]), 51.67% of) the thickness of the lens measured in each MRI. Since Hughes’s model was originally developed with young adult rats, these modifications were used here and in previous work [4] to scale the model for age-related changes in eye/lens size. A final consideration in refractive state calculations is the position where incoming light is focused: the photopigment-laden disks within the rod outer segments. For these calculations, retinal thicknesses are measured from MRI images as described in the main text, and the position of disks is estimated at 84% of that thickness (‘84% thick’) beyond the vitreoretinal border: Recent *in vivo* optical coherence tomography (OCT) images of Long-Evans rat retinas [6]–[8] localize the bacillary layer (rod inner and outer segments) between ∼71% thick and ∼93% thick, and histologic studies show that inner segments are ∼2/3 rds the length of outer segments throughout adulthood [9]–[14]. Considering the present data alongside previous reports [4], [15]–[17], it seems that rats are myopic (near-sighted; refractive state <0 Diopters) just after their eyes open (age ∼ 14 d), but rapidly shift from myopia to emmetropia (0 D) in the subsequent 2 mo of life. Somewhere near age 3 mo, rats transition to hyperopia (>0 D), then show relatively small (but statistically significant) increases in refractive state in the following 15 mo of life. *Scatter Plots (Retinal Morphology):* The retina significantly thins with age – a pattern which may partially be due to stretching of the retina as the eye continues to grow [18]. Consistent with that analysis, and with previous work in both humans and rats [18]–[21], we found significant age-related increases in retinal extent and surface area. Also, total retinal volume increased with age, arguing against the possibility that thinning is representative of cell loss. *Scatter Plots (Mn^2+^ Uptake in the Inner Retina):* These plots of data from the *inner* retina (interior to the photoreceptors, at 16–28% thick, corresponding to the inner plexiform layer) complement the corresponding plots from the *outer* retina (photoreceptors) shown in main text Figure 3. The ΔR_1_s shown here were calculated after subtracting the Mn^2+^-enhanced R_1_s from the average *inner retinal* baseline R_1_ – i.e., *without* Mn^2+^ enhancement – of 0.514 s^−1^. That baseline was not affected by light or age (*P*>0.4), with mean±SEM R_1_s of 0.52±0.03 s^−1^ (young 0.58±0.04 s^−1^; mid 0.44±0.03 s^−1^; old 0.54±0.04 s^−1^) in patched eyes, and 0.51±0.02 s^−1^ (young 0.53±0.03 s^−1^; mid 0.51±0.03 s^−1^; old 0.48±0.07 s^−1^) in unpatched eyes. Inner retinal Mn^2+^ uptake (ΔR_1_) increased significantly with age in both Group YM and Group MO, and in both darkness and light. In contrast to the outer retina, the inner retina showed no dark-light differences at any age (all tests for differences >0, and for ratios >1, yielded *P*>0.05), consistent with previous work [22]–[24]. For this reason, dark and light *inner* retinal data (from a given rat at a given time point) were averaged prior to the regression analyses featured in Tables S1 through S4. Finally, we note that a single young adult dark/light ratio value of 4.69 was retained in statistical comparisons, but was omitted from this dark/light ratio plot so as to retain a useful y-axis range (this is the only scatter plot omission throughout the entire present work). **Figure S4, related to** **Figure 4:** **Visualization of regression results linking initial Mn^2+^ uptake (and optics/eye size) measurements to subsequent CS declines.** In these plots, groups are split based on whether an animal’s first measurement (at age ∼2.5 mo for Group YM (circles); at age ∼7 mo for Group MO (squares)) was higher or lower than the median value for that group at the time of the first measurement. Note that these splits are intended for visualization purposes only: Because distributions were roughly normal in all cases, these high/low categories were *not* used in formal statistical comparisons, which were performed with linear regression (see Tables S1–S4). Only animals which were studied longitudinally (i.e., vision tested ∼4.5 mo after the first measurement) were used to calculate medians, or the mean±SEM plotted for each sub-group (note that, for visual clarity, the lower right panel omits error bars). Note that the occasionally large span covered by SEM error bars arises from baseline inter-individual differences, which have little influence on our formal analyses that are based on pair-wise longitudinal comparisons. Retinal surface area is representative of most of the other morphological measures (including those concerning optics/eye size): Its starting values and rates of change were strongly correlated with starting values and rates of change for most other eye size measures (Tables S1, S3, S4). *The critical features of these plots are the slopes of connecting lines, which represent the rates of change described in Table S4*. *Top Left*: Rats starting with relatively (for their age) low surface area experience significantly greater growth over the course of the study (steeper slopes on dashed than solid lines; r values <0 in Table S4, pt.2) but still end our studies with relatively lower surface area (r values >0 in Table S2) than other cohort members. *Middle Left:* There are no consistent relationships between starting surface area and starting values of, or rates of change in, retinal Mn^2+^ uptake (q>0.05 in Tables S1, S4). *Bottom Left:* Measuring from the first to the final time point for each group, rats starting with higher surface areas experience significantly greater CS declines (steeper (more negative) slopes on solid than dotted lines; r values <0 in Table S4). Note, however, that the magnitude of this relationship is small compared to the relationship between CS declines and Mn^2+^ uptake shown in the remaining panels. *Middle Center*: Rats starting with relatively low Mn^2+^ uptake experience significantly greater increases in Mn^2+^ uptake over the course of the study (steeper slopes on orange than purple; Table S4, pt.2). Levels of Mn^2+^ uptake converge so much over time that starting and ending values are not correlated (q>0.05 in Table S2). *Bottom Center*: In the ∼4.5 mo after MRI scans, rats starting with high Mn^2+^ uptake showed significantly steeper CS declines (steeper slopes for purple than orange; Table S4, pt.1), but from ∼4.5 to ∼12 mo after MRI scans, the opposite pattern was seen in Group MO (squares). *Bottom Right:* The patterns at Bottom Left and Bottom Center are present when splitting data by both surface area and Mn^2+^ uptake, arguing that their relationships to contrast sensitivity changes are largely independent of one-another. In Group MO (squares), ultimate (over 1 year) CS declines are predictable by starting surface area (solid lines steeper than dashed), but the tendency to decline immediately (within ∼4.5 mo of scan; purple lines) versus retain function until a later eventual decline (from ∼4.5 mo to ∼12 mo; orange lines) is predicted by starting Mn^2+^ uptake, regardless of starting surface area. **Figure S5, related to** **Figure 5:** ***Inner*** **retinal Mn^2+^ uptake (ΔR_1_; in s^−1^) in nifedipine-treated and vehicle control eyes.** These data correspond to the *outer* retinal data shown in main text Figure 5. Paired measurements (left and right eye of each rat) are connected by grey lines in the top panels, and subtracted from one-another to produce the bottom panels. *Left:* As expected for the inner retina (see Fig. S3 and legend), no dark-light differences are noted in either group. Mn^2+^ uptake non-significantly trended lower in nifedipine-injected than in vehicle-control rats, for both light-exposed and dark-adapted (patched) inner retina (*P* = 0.128 and 0.0573, respectively). When assessed with a two-way (light vs. dark × drug vs. vehicle) mixed ANOVA, the drug effect was suggestive (F[_1,12_] = 3.15; *P* = 0.101) but none of the ANOVA results reached significance (F_[1, 12]_<0.59; *P*>0.45 for both the light effect and interaction). *Right:* Exposure to nifedipine eye drops failed to have a significant effect on Mn^2+^ uptake in the dark-adapted *inner* retina (*P = *0.379). *Combined Analysis of Both Experiments:* In contrast to the positive results noted for the outer retina (main text Fig. 5) in both experiments, neither experiment yielded a significant effect of nifedipine on *inner* retinal Mn^2+^ uptake. It is plausible that the relatively small sample sizes used in each experiment contributed to these negative findings. We therefore combined the results with a meta-analysis technique, since both datasets are used to test the same general hypothesis – that nifedipine decreases *inner* retinal Mn^2+^ uptake. We used the Z-transform method, weighted by degrees of freedom (‘df’) (Whitlock, 2005), to combine the ANOVA main effect for drug (*P = *0.101; df = 12) in the systemic nifedipine experiment with the t-test comparing control and nifedipine eye drops (*P = *0.379; df = 4). Consistent with expectations, the combined results show significant (*P = *0.0493) inhibition of *inner* retinal Mn^2+^ uptake by nifedipine. Reassuringly, measurements with a different L-VGCC blocker (diltiazem; Fig. S6) support this finding. **Figure S6, related to** **Figure 7:** **Inner and outer retinal data show an age-related change in sensitivity to diltiazem.** *Top:* Data from both the inner retina (*Left*) and outer retina (*Right*) are shown in scatter plots, with best-fit lines (±95% C.I.) for young and mid-adults in the background. Note that two pairs of mid-adult data points for the *outer* retina overlap: one pair at 100 mg/kg (ΔR_1_s of 0.471 and 0.475) and one at 125 mg/kg (ΔR_1_s of 0.551 and 0.556). Progressively higher doses of diltiazem are correlated with progressively less Mn^2+^ uptake (i.e., greater inhibition; *P*<0.05) in mid-adults, but have little additional effects in young adults (*P*>0.05). Multiple regression analyses run on the inner and outer retinal data confirm the impression that mid-adults generally have greater Mn^2+^ uptake than young adults (*P*<1.3e-4), that Mn^2+^ uptake is generally lower with higher doses of diltiazem (*P*<0.02), and that mid-adults have a steeper dose-response curve than young-adults (*P*<0.04). Based on the analysis in the bottom half of this figure, it seems that the dose-response curve is essentially flat in young adults because – *in that age group* – diltiazem appears to exert its maximum effect even at a dose of 10 mg/kg. This is in reasonable agreement with our previous study of young adult rats, which showed substantial inhibition of Mn^2+^ uptake even with a 5 mg/kg dose [25]. In contrast to the young adults, mid-adults require much higher doses of diltiazem to produce the expected drug effects. *Bottom:* These data from the *inner* retina are presented in the same style as the corresponding outer retinal data from main text Fig. 7. Mn^2+^ uptake in these diltiazem-injected dark-adapted rats is compared to the age-matched dark-adapted control data from our longitudinal studies (see main text Fig. 3). The right y-axis for each plot shows % inhibition, calculated as [1– (ΔR_1,diltiazem/_ΔR_1,control_)]. In young adults, diltiazem significantly inhibits Mn^2+^ uptake (*P<0.05) at both low and high doses: Beyond the ∼40% inhibition seen with the low dose, no additional inhibition is seen with high doses (‘n.s.’ indicates not significant; *P*>0.05). In mid-adults, low doses of diltiazem showed no effect on Mn^2+^ uptake, but high doses yielded significant (*P<0.05; ∼40%) inhibition of Mn^2+^ uptake. As discussed in the main text, these data suggest that there is an age-related decrease in sensitivity to L-VGCC blockers, though with high (and not clinically relevant) doses, maximum inhibition is still possible. This pattern is consistent with an age-related increase in retinal expression of the relatively drug-insensitive α_1D_ L-VGCC isoform.
(DOC)
