# Supplementary material for: Diminished Vision in Healthy Aging Is Associated with Increased Retinal L-Type Voltage Gated Calcium Channel Ion Influx
Source: PLoS One. 2013 Feb 14;8(2):e56340. doi: 10.1371/journal.pone.0056340 (PMC3572962; doi:10.1371/journal.pone.0056340)
Supplement: File S1 — This file contains the following figures: Figure S1, related to Figure 1: Details of the in-house-built OKT device. The device was made of three identical 19′′ LCD monitors arranged in an equilateral triangle. Mirrors placed on the floor and ceiling reflected a moving sine wave grating, displayed and distorted identically on each screen with Vision Egg (v.1.0), thereby forming a virtual barrel (i.e., the width of one dark-light-dark cycle appearing similar in all directions) when viewed from the center of the device. A: An overhead view with screen faces represented by solid black lines, and screen edges connected by dashed lines (the corners of the device are occupied by screen casings, which are not shown in this panel). The rat’s right eye is shown just inside of the bulging-triangle shape that defines the testing arena. Tracking was recorded only if the rat remained on the perch and the stimulated eye (left eye if the virtual barrel was moving clockwise; right if counter-clockwise) was inside the arena. The arena was marked only on the operator’s screen, superimposed on and calibrated to the overhead video feed (Microsoft LifeCam VX-2000). Azimuth – direction in the horizontal plane with 0° at the center of the lower left screen – is used as the x-axis for panel C. B: Side view, with one screen removed. The perch was constructed of a single piece of thick metal wire, padded at the hind- and forelimb placements, and secured at the center and one corner of the device floor. C: As a rat’s eye moves closer to a screen, less of the nearest dark-light-dark cycle fits within a degree visual angle. Measuring from the position of the rat’s left eye in panel A, and a device setting of 0.111 c/bd (used for all CS measurements), stimuli at 0° azimuth will have the spatial frequency of 0.091 cycles per degree visual angle. This is plotted as a cycles-per-degree-visual-angle to cycles-per-barrel-degree ratio of 0.82( = 0.091/0.111). Near the corners of the device (gaps at −60 [file pone.0056340.s001.doc]

**
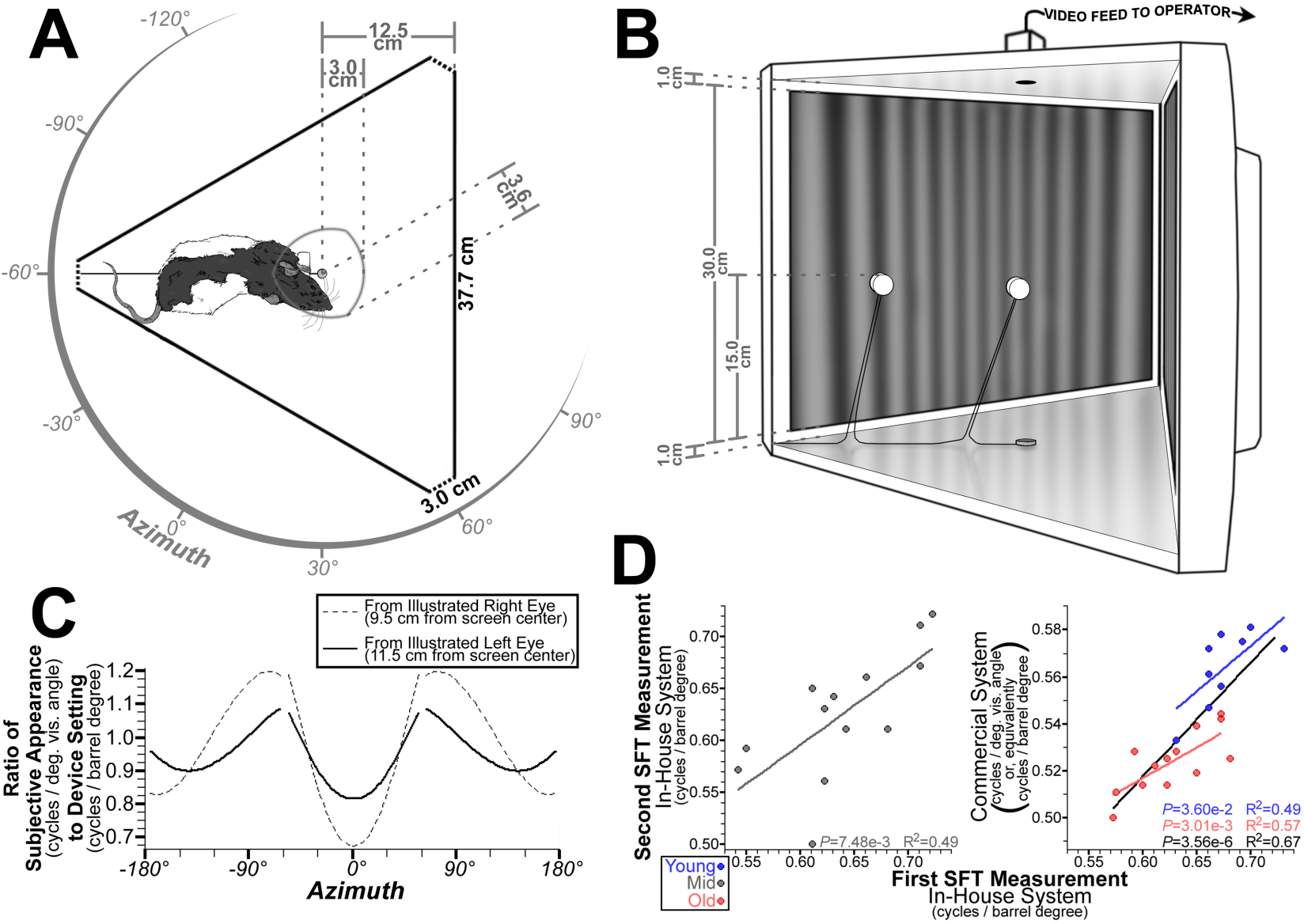
 Figure S1, related to Figure 1: Details of the in-house-built OKT device**.

**
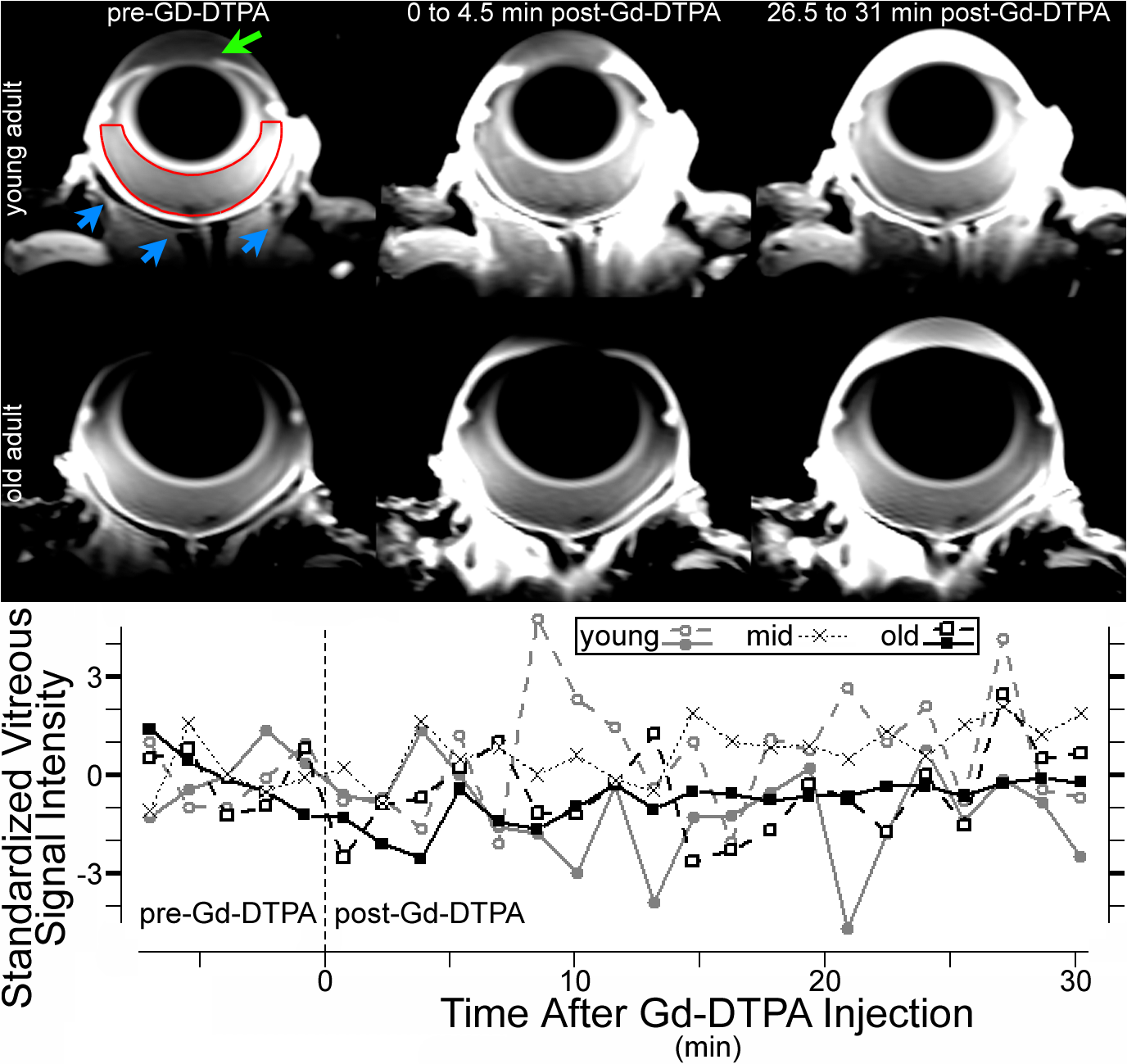
**

**Figure S2, related to Figure 2: The blood-retinal barrier (BRB) is intact throughout adulthood.**

**
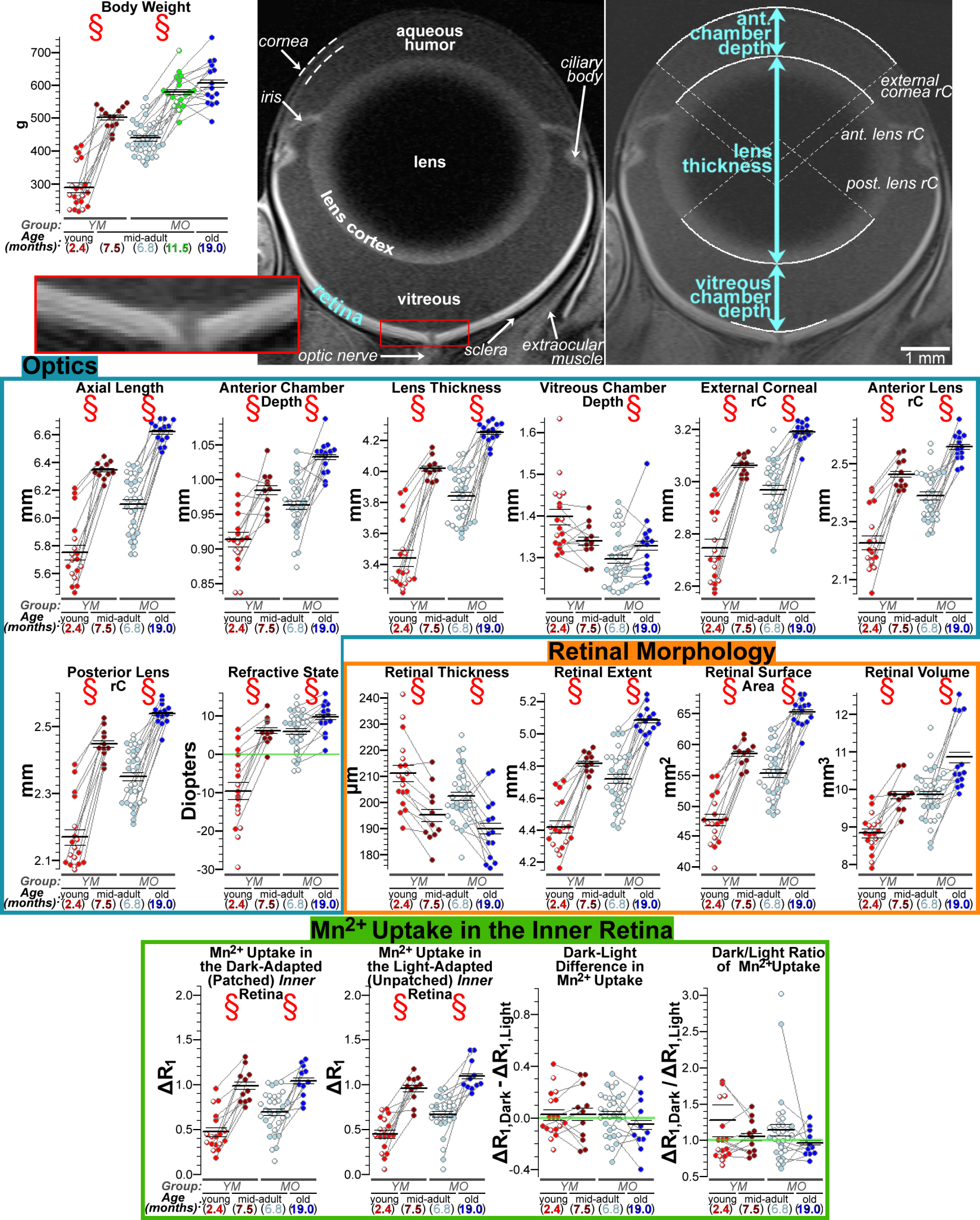
**

**Figure S3, related to Figure 3: Longitudinal analyses of morphology and inner retinal Mn^2+^ uptake.**

**Table S1, related to Figure 4:** **Correlations between measurements at the first time point in our longitudinal studies (at ~2.5 mo in Group YM, and ~7 mo in Group MO).** Correlation coefficients (Pearson’s r) are listed for each analysis as [(Group YM , Group MO) Combined]. Bold indicates significance (q<0.05; FDR calculated on 205 tests): When an r value is listed for the combined analysis, group-specific r values are shown only for completeness. When * appears instead of an r value, an interaction between group and the correlation was suspected (*P* < 0.05) and statistics were run on each group separately. In general initial values for both vision tests (SFT and CS) are poorly-correlated with morphological measures (including sizes of optical components and retinal morphology) and measures of Mn^2+^ uptake. Morphological measures are well-correlated with one-another. Measures of Mn^2+^ uptake are reasonably well-correlated with one-another. However, morphological and Mn^2+^ uptake measures generally are poorly-correlated.

|  | Body Weight | SFT | CS | Ant. Chamber Depth | Lens Thickness | Vit. Chamber Depth | Axial Length | Corneal rC | Anterior Lens rC | Posterior Lens rC | Refractive State | Retinal Thickness | Retinal Extent | Retinal Surface Area | Retinal Volume | ΔR_1_, (inner retina) | ΔR^1,Dark^ (outer retina) | ΔR_1,Light_  (outer retina) | ΔR_1,Dark_ - ΔR_1,Light_ (outer retina) |
| --- | --- | --- | --- | --- | --- | --- | --- | --- | --- | --- | --- | --- | --- | --- | --- | --- | --- | --- | --- |
| ΔR_1,Dark_ / ΔR_1,Light_ (outer retina) | (0.3,0.1)  0.16 | (-0.5,-0.2)  -0.28 | (0.0,0.2)  0.13 | (0.2,-0.1)  0.00 | (0.2,0.2)  0.19 | (-0.2,0.1)  -0.03 | (0.2,0.1)  0.15 | (0.2,0.0)  0.09 | (0.2,0.1)  0.16 | (0.2,0.2)  0.22 | (0.2,0.0)  0.08 | (-0.5,-0.2)  -0.28 | (0.2,0.0)  0.08 | (0.1,0.0)  0.03 | (-0.2,0.0)  -0.07 | (0.2,0.2)  0.16 | (0.4,0.5)  **0.49** | (-0.3,-0.3)  -0.31 | (0.9,0.9)  **0.92** |
| ΔR_1,Dark_ - ΔR_1,Light_ (outer retina) | (0.6,0.1)  0.28 | (-0.3,-0.2)  -0.23 | (0.0,0.2)  0.13 | (0.4,0.0)  0.13 | (0.5,0.2)  0.30 | (-0.4,0.0)  -0.13 | (0.4,0.1)  0.24 | (0.4,0.1)  0.18 | (0.5,0.2)  0.31 | (0.5,0.3)  **0.36** | (0.5,0.0)  0.21 | (-0.6,-0.2)  **-0.37** | (0.3,0.0)  0.13 | (0.3,0.0)  0.10 | (-0.1,0.0)  -0.01 | (0.5,0.4)  **0.41** | (0.7,0.7)  **0.73** | (0.0,-0.1)  -0.05 |  |
| ΔR_1,Light_ (outer retina) | (0.6,0.2)  **0.33** | (0.5,-0.1)  0.13 | (0.5,-0.2)  * | (0.3,0.1)  0.12 | (**0.6**,0.0)  * | (-0.2,-0.2)  -0.18 | (0.5,-0.1)  0.15 | (0.5,0.0)  0.18 | (0.6,0.1)  0.26 | (**0.6**,0.0)  * | (0.4,0.2)  0.26 | (-0.2,-0.3)  -0.25 | (0.5,0.0)  0.17 | (0.5,0.0)  0.18 | (0.5,0.0)  0.16 | (0.9,0.8)  **0.79** | (0.7,0.6)  **0.64** |  |  |
| ΔR_1,Dark_ (outer retina) | (**0.8**,0.2)  * | (0.1,-0.2)  -0.07 | (0.4,0.0)  0.14 | (0.5,0.0)  0.17 | (**0.8**,0.1)  * | (-0.4,-0.1)  -0.21 | (**0.7**,0.1)  * | (0.6,0.1)  0.25 | (0.7,0.2)  **0.41** | (**0.8**,0.2)  * | (0.6,0.1)  **0.32** | (-0.6,-0.4)  **-0.44** | (**0.6**,0.0)  * | (**0.6**,0.0)  * | (0.3,0.0)  0.11 | (0.9,0.8)  **0.86** |  |  |  |
| ΔR_1_, (inner retina) | (**0.8**,0.2)  * | (0.2,-0.2)  -0.07 | (0.4,-0.2)  0.03 | (0.5,0.0)  0.19 | (**0.7**,0.1)  * | (-0.4,-0.2)  -0.27 | (**0.6**,0.0)  * | (0.5,0.0)  0.20 | (0.7,0.2)  **0.38** | (**0.8**,0.1)  * | (0.5,0.2)  **0.32** | (-0.4,-0.1)  -0.22 | (0.6,0.0)  0.21 | (**0.6**,0.0)  * | (0.4,0.1)  0.19 |  |  |  |  |
| Retinal Volume | (0.6,0.7)  **0.65** | (0.3,0.0)  0.09 | (0.4,0.0)  0.13 | (0.1,0.4)  **0.33** | (0.6,0.8)  **0.72** | (0.2,0.1)  0.17 | (0.7,0.8)  **0.73** | (0.5,0.8)  **0.68** | (0.7,0.8)  **0.73** | (0.6,0.6)  **0.60** | (0.1,0.4)  0.27 | (0.1,0.1)  0.09 | (0.7,0.8)  **0.79** | (0.5,0.8)  **0.73** |  |  |  |  |  |
| Retinal Surface Area | (0.8,0.8)  **0.79** | (0.3,-0.1)  0.04 | (0.5,-0.1)  0.14 | (0.6,0.6)  **0.61** | (0.8,0.9)  **0.85** | (-0.5,0.0)  -0.16 | (0.7,0.8)  **0.78** | (0.8,0.8)  **0.82** | (0.9,0.8)  **0.84** | (0.8,0.7)  **0.73** | (0.8,0.5)  **0.61** | (-0.7,-0.3)  **-0.44** | (0.9,1.0)  **0.96** |  |  |  |  |  |  |
| Retinal Extent | (0.8,0.8)  **0.81** | (0.2,-0.2)  -0.01 | (0.5,0.0)  0.16 | (0.6,0.6)  **0.56** | (0.9,0.9)  **0.87** | (-0.2,0.2)  0.01 | (0.8,0.9)  **0.85** | (0.8,0.8)  **0.84** | (0.9,0.8)  **0.85** | (0.8,0.7)  **0.74** | (0.7,0.4)  **0.50** | (-0.6,-0.3)  **-0.38** |  |  |  |  |  |  |  |
| Retinal Thickness | (-0.5,-0.4)  **-0.42** | (0.0,0.0)  -0.03 | (-0.2,0.1)  -0.01 | (-0.6,-0.3)  **-0.40** | (-0.6,-0.3)  **-0.36** | (**0.7**,0.1)  * | (-0.4,-0.2)  -0.29 | (-0.6,-0.3)  **-0.38** | (-0.6,-0.3)  **-0.38** | (-0.6,-0.3)  **-0.38** | (-0.8,-0.3)  **-0.52** |  |  |  |  |  |  |  |  |
| Refractive State | (0.7,0.4)  **0.48** | (0.1,-0.1)  -0.07 | (0.4,-0.2)  0.02 | (0.6,0.4)  **0.49** | (0.8,0.5)  **0.60** | (-0.8,-0.8)  **-0.76** | (0.5,0.3)  **0.36** | (0.8,0.6)  **0.65** | (0.7,0.5)  **0.60** | (0.8,0.4)  **0.56** |  |  |  |  |  |  |  |  |  |
| Posterior Lens rC | (0.9,0.8)  **0.80** | (0.1,-0.4)  -0.19 | (0.5,0.2)  **0.34** | (0.6,0.6)  **0.58** | (1.0,0.9)  **0.95** | (-0.4,0.1)  -0.05 | (0.9,0.9)  **0.90** | (0.9,0.9)  **0.88** | (0.9,0.9)  **0.92** |  |  |  |  |  |  |  |  |  |  |
| Anterior Lens rC | (0.9,0.8)  **0.85** | (0.2,-0.1)  -0.01 | (0.5,0.1)  0.22 | (0.6,0.6)  **0.58** | (1.0,1.0)  **0.96** | (-0.3,0.1)  -0.05 | (0.9,0.9)  **0.91** | (0.9,0.9)  **0.92** |  |  |  |  |  |  |  |  |  |  |  |
| Corneal rC | (0.8,0.8)  **0.75** | (0.0,-0.2)  -0.11 | (0.5,0.1)  0.21 | (0.4,0.6)  **0.52** | (0.9,1.0)  **0.94** | (-0.2,0.1)  -0.03 | (0.9,0.9)  **0.89** |  |  |  |  |  |  |  |  |  |  |  |  |
| Axial Length | (0.8,0.8)  **0.83** | (0.1,-0.2)  -0.12 | (0.5,0.2)  0.31 | (0.6,0.6)  **0.61** | (1.0,0.9)  **0.95** | (0.0,0.4)  0.23 |  |  |  |  |  |  |  |  |  |  |  |  |  |
| Vit. Chamber Depth | (-0.4,0.1)  -0.03 | (-0.1,0.1)  0.05 | (-0.1,0.2)  0.10 | (-0.5,-0.1)  -0.26 | (-0.3,0.1)  -0.04 |  |  |  |  |  |  |  |  |  |  |  |  |  |  |
| Lens Thickness | (0.9,0.9)  **0.87** | (0.1,-0.3)  -0.12 | (0.6,0.1)  0.28 | (0.6,0.6)  **0.60** |  |  |  |  |  |  |  |  |  |  |  |  |  |  |  |
| Ant. Chamber Depth | (0.6,0.5)  **0.53** | (0.1,-0.4)  -0.22 | (0.3,0.1)  0.15 |  |  |  |  |  |  |  |  |  |  |  |  |  |  |  |  |
| CS | (0.5,0.2)  **0.33** | (0.1,-0.1)  0.02 |  |  |  |  |  |  |  |  |  |  |  |  |  |  |  |  |  |
| SFT | (0.0,-0.2)  -0.12 |  |  |  |  |  |  |  |  |  |  |  |  |  |  |  |  |  |  |

**Table S2, related to Figure 4: Correlations between initial and later measurements of each variable.** Correlation coefficients (Pearson’s r) values are listed for each comparison. Bold indicates significance (q < 0.05; FDR calculated on 26 tests — twenty of the combined data and six with the ~11.5 mo age as a start or endpoint). Formal analyses combined z-standardized Group YM and Group MO data. For completeness, r values calculated for each group are also shown. Significant findings here, with positive r values, indicate that rats which had larger-than-average eyes (represented by axial length, etc.) at the start of the study also had larger-than-average eyes at the end of the study. This is despite some convergence of values over time — a pattern clearly visible for e.g. axial length in Figure S3, or retinal surface area in Figure S4, and noted statistically by the significant inverse relationships between a variable’s initial measurement and subsequent rate of change (Table S4). This convergence is even more substantial for Mn^2+^ uptake (ΔR_1_s) data (see also Fig.S4), resulting in the negative findings in this table.

|  |  | **From the start to the end of the experiment** | | | **With ~11.5 mo data as start or endpoint** | |
| --- | --- | --- | --- | --- | --- | --- |
|  |  |  |  |  |  |  |
| Group | | **YM** | **MO** | **Combined** | **MO** | |
| Age Range (months) | | ~2.5 to ~7 | ~7 to ~19 | ← | ~7 to ~11.5 | ~11.5 to ~19 |
| Body Weight | | (n=13) | (n=15) | (n=28) | (n=19) | (n=15) |
|  |  | 0.05 | 0.41 | 0.25 | **0.85** | **0.60** |
| Behavior |  | (n=13) | (n=15) | (n=28) | (n=19) | (n=15) |
|  | STF | 0.77 | 0.59 | **0.67** | 0.37 | **0.68** |
|  | CS | 0.11 | 0.06 | 0.09 | 0.40 | 0.37 |
| Morphology and Optics |  | (n=11) | (n=14) | (n=25) |  |  |
|  | Ant. Chamber Depth | 0.08 | 0.05 | 0.06 |  |  |
|  | Lens Thickness | 0.90 | 0.77 | **0.83** |  |  |
|  | Vit. Chamber Depth | 0.52 | 0.51 | **0.52** |  |  |
|  | Axial Length | 0.72 | 0.53 | **0.61** |  |  |
|  | Corneal rC | 0.83 | 0.68 | **0.75** |  |  |
|  | Anterior Lens rC | 0.84 | 0.37 | **0.57** |  |  |
|  | Posterior Lens rC | 0.76 | 0.39 | **0.55** |  |  |
|  | Refractive State | 0.80 | 0.53 | **0.65** |  |  |
|  | Retinal Thickness | 0.80 | 0.08 | 0.39 |  |  |
|  | Retinal Extent | 0.73 | 0.60 | **0.66** |  |  |
|  | Retinal Surface Area | 0.74 | 0.62 | **0.67** |  |  |
|  | Retinal Volume | 0.55 | 0.12 | 0.31 |  |  |
| Physiology |  | (n=11) | (n=11) | (n=22) |  |  |
|  | ΔR_1_ (inner retina) | 0.29 | 0.01 | 0.15 |  |  |
|  | ΔR_1,Dark_ (outer retina) | 0.39 | 0.12 | 0.26 |  |  |
|  | ΔR_1,Light_ (outer retina) | 0.11 | 0.61 | 0.36 |  |  |
|  | ΔR_1,Dark_ - ΔR_1,Light_ (outer retina) | 0.31 | 0.52 | 0.42 |  |  |
|  | ΔR_1,Dark_ / ΔR_1,Light_ (outer retina) | 0.04 | 0.48 | 0.26 |  |  |

**Table S3, related to Figure 4**: **Correlations between longitudinal rate of change in one variable with rate of change in another.** Correlation coefficients (Pearson’s r) are listed for each analysis as [(Group YM , Group MO) Combined]. Bold indicates significance (q < 0.05; FDR calculated on 206 tests): When r is listed for the combined analysis, group-specific r values are shown only for completeness. Where an * appears instead of an r value, an interaction between group and the correlation was suspected (*P* < 0.05) and statistics were run on each group separately. Additional tests (included in the 206 total) using Group MO’s age ~11.5 mo data as a start or endpoint (body weight vs. SFT, body weight vs. CS, and SFT vs. CS) all produced negative results (-0.31 < r < 0.27; P > 0.2; not shown). We generally find strong positive correlations between measures of eye size (e.g. axial length) and retinal morphology (e.g. surface area) — consistent with well-coordinated eye growth throughout adulthood. Interestingly, the rate of change in surface area is poorly-correlated with the rate of change in retinal thickness. Rates of change in morphology are generally poorly-correlated with rates of change in Mn^2+^ uptake. However, rates of change in Mn^2+^ uptake are reasonably well-correlated with one-another.

|  | Body Weight | SFT | CS | Ant. Chamber Depth | Lens Thickness | Vit. Chamber Depth | Axial Length | Corneal rC | Anterior Lens rC | Posterior Lens rC | Refractive State | Retinal Thickness | Retinal Extent | Retinal Surface Area | Retinal Volume | ΔR_1_, (inner retina) | ΔR_1,Dark_ (outer retina) | ΔR_1,Light_ (outer retina) | ΔR_1,Dark_ - ΔR_1,Light_ (outer retina) |
| --- | --- | --- | --- | --- | --- | --- | --- | --- | --- | --- | --- | --- | --- | --- | --- | --- | --- | --- | --- |
| ΔR_1,Dark_ / ΔR_1,Light_ (outer retina) | (0.4,0.2)  0.28 | (0.0,-0.1)  -0.05 | (0.0,0.0)  0.02 | (0.2,-0.4)  -0.12 | (0.1,0.2)  0.16 | (0.3,0.2)  0.25 | (0.2,0.1)  0.14 | (-0.2,0.1)  -0.07 | (0.0,0.0)  0.01 | (0.1,0.0)  0.06 | (-0.4,-0.3)  -0.34 | (-0.4,0.7)  * | (0.0,-0.1)  -0.05 | (-0.1,-0.3)  -0.22 | (-0.3,0.1)  -0.11 | (-0.1,0.1)  0.01 | (0.5,0.5)  0.5 | (-0.4,-0.2)  -0.33 | (0.9,0.8)  **0.85** |
| ΔR_1,Dark_ - ΔR_1,Light_ (outer retina) | (0.2,0.0)  0.10 | (0.3,0.2)  0.23 | (0.0,0.0)  -0.02 | (0.2,-0.5)  -0.13 | (0.1,0.1)  0.11 | (0.3,0.3)  0.26 | (0.2,0.0)  0.11 | (-0.3,0.1)  -0.08 | (0.0,0.1)  0.04 | (0.1,-0.1)  -0.01 | (-0.4,-0.3)  -0.36 | (-0.2,**0.8**)  * | (-0.1,-0.1)  -0.08 | (-0.2,-0.3)  -0.25 | (-0.3,0.1)  -0.09 | (0.1,0.4)  0.23 | (0.7,0.7)  **0.70** | (-0.3,-0.2)  -0.23 |  |
| ΔR_1,Light_ (outer retina) | (0.5,0.3)  0.38 | (-0.2,-0.1)  -0.13 | (0.6,0.0)  0.29 | (0.6,0.2)  0.37 | (0.7,0.0)  0.35 | (0.2,-0.5)  -0.15 | (0.7,-0.1)  0.29 | (0.7,-0.1)  0.28 | (0.7,0.0)  0.35 | (0.7,0.0)  0.36 | (0.6,0.2)  0.37 | (0.0,0.0)  -0.01 | (0.8,-0.1)  0.34 | (0.8,-0.0)  0.37 | (0.6,-0.2)  0.19 | (0.9,0.6)  **0.73** | (0.4,0.6)  **0.53** |  |  |
| ΔR_1,Dark_ (outer retina) | (0.5,0.2)  0.37 | (0.1,0.1)  0.10 | (0.4,-0.1)  0.18 | (0.6,-0.2)  0.18 | (0.6,0.1)  0.35 | (0.4,-0.2)  0.10 | (0.7,-0.1)  0.30 | (0.2,0.0)  0.10 | (0.5,0.0)  0.28 | (0.6,-0.1)  0.26 | (0.0,-0.1)  -0.05 | (-0.2,0.6)  0.20 | (0.5,-0.1)  0.17 | (0.4,-0.3)  0.05 | (0.1,0.0)  0.03 | (0.7,0.7)  **0.72** |  |  |  |
| ΔR_1_, (inner retina) | (0.6,-0.1)  0.24 | (-0.1,0.3)  0.10 | (0.7,0.0)  0.34 | (**0.7**,-0.2)  * | (**0.9**,-0.3)  * | (0.1,-0.3)  -0.12 | (**0.8**,-0.4)  * | (0.7,-0.2)  * | (**0.8**,-0.2)  * | (**0.8**,-0.4)  * | (0.5,0.1)  0.30 | (-0.1,0.2)  0.06 | (**0.8**,-0.2)  * | (**0.8**,-0.2)  * | (0.5,-0.1)  0.23 |  |  |  |  |
| Retinal Volume | (0.4,0.5) 0.45 | (0.3,0.0) 0.12 | (0.5,0.4) 0.46 | (0.3,0.6) 0.46 | (0.6,0.7) **0.69** | (0.1,0.2) 0.15 | (0.6,0.7) **0.64** | (0.6,0.7) **0.66** | (0.7,0.3) 0.43 | (0.7,0.6) **0.65** | (0.5,0.1) 0.29 | (0.5,0.6) **0.60** | (0.5,0.8) **0.65** | (0.3,0.7)**0.53** |  |  |  |  |  |
| Retinal Surface Area | (0.7,0.5)  **0.60** | (-0.3,-0.1)  -0.22 | (0.7,0.6)  **0.61** | (0.8,0.8)  **0.79** | (0.8,0.8)  **0.80** | (-0.1,0.1)  0.02 | (0.8,0.8)  **0.77** | (0.7,0.8)  **0.78** | (0.7,0.6)  **0.64** | (0.7,0.7)  **0.72** | (0.6,0.4)  **0.52** | (-0.4,0.2)  -0.05 | (0.9,0.9)  **0.94** |  |  |  |  |  |  |
| Retinal Extent | (0.8,0.5)  **0.61** | (-0.2,0.0)  -0.09 | (0.7,0.5)  **0.58** | (0.8,0.7)  **0.77** | (0.9,0.9)  **0.91** | (0.1,0.1)  0.10 | (0.9,0.8)  **0.86** | (0.8,0.9)  **0.82** | (0.9,0.5)  **0.68** | (0.9,0.7)  **0.78** | (0.6,0.4)  **0.49** | (-0.3,0.3)  0.05 |  |  |  |  |  |  |  |
| Retinal Thickness | (-0.2,0.2)  0.02 | (0.5,-0.1)  0.18 | (0.2,-0.1)  0.02 | (-0.4,0.2)  -0.06 | (-0.1,0.5)  0.23 | (0.1,0.4)  0.27 | (-0.1,0.5)  0.22 | (0.1,0.3)  0.21 | (0.0,0.1)  0.04 | (0.1,0.3)  0.22 | (0.0,-0.5)  -0.25 |  |  |  |  |  |  |  |  |
| Refractive State | (0.4,0.2)  0.32 | (-0.3,0.2)  -0.02 | (0.7,0.3)  0.47 | (0.3,0.2)  0.26 | (0.7,0.2)  0.39 | (-0.4,-0.7)  **-0.54** | (0.5,-0.1)  0.18 | (0.9,0.3)  **0.57** | (0.6,0.2)  0.40 | (0.6,0.2)  0.37 |  |  |  |  |  |  |  |  |  |
| Posterior Lens rC | (0.8,0.7)  **0.75** | (0.0,0.0)  0.02 | (0.8,0.5)  **0.63** | (0.7,0.8)  **0.75** | (1.0,0.9)  **0.93** | (0.3,0.3)  0.28 | (1.0,0.9)  **0.92** | (0.8,0.8)  **0.83** | (0.9,0.8)  **0.86** |  |  |  |  |  |  |  |  |  |  |
| Anterior Lens rC | (0.7,0.6)  **0.65** | (0.2,0.2)  0.16 | (0.7,0.5)  **0.57** | (0.7,0.6)  **0.67** | (0.9,0.7)  **0.77** | (0.3,0.4)  0.34 | (0.9,0.7)  **0.82** | (0.8,0.8)  **0.81** |  |  |  |  |  |  |  |  |  |  |  |
| Corneal rC | (0.7,0.7)  **0.69** | (-0.2,0.2)  0.01 | (0.8,0.6)  **0.68** | (0.4,0.8)  **0.60** | (0.8,0.8)  **0.84** | (0.1,0.4)  0.26 | (0.8,0.9)  **0.82** |  |  |  |  |  |  |  |  |  |  |  |  |
| Axial Length | (0.8,0.6)  **0.72** | (0.0,0.0)  -0.01 | (0.7,0.5)  **0.58** | (0.8,0.8)  **0.80** | (1.0,0.9)  **0.94** | (0.3,0.6)  0.46 |  |  |  |  |  |  |  |  |  |  |  |  |  |
| Vit. Chamber Depth | (0.3,0.2)  0.26 | (0.0,0.0)  0.04 | (0.1,0.1)  0.11 | (-0.1,0.3)  0.12 | (0.1,0.3)  0.20 |  |  |  |  |  |  |  |  |  |  |  |  |  |  |
| Lens Thickness | (0.8,0.6)  **0.71** | (0.0,-0.1)  -0.06 | (0.8,0.4)  **0.59** | (0.8,0.7)  **0.73** |  |  |  |  |  |  |  |  |  |  |  |  |  |  |  |
| Ant. Chamber Depth | (0.6,0.6)  **0.61** | (0.1,0.0)  0.08 | (0.5,0.5)  **0.50** |  |  |  |  |  |  |  |  |  |  |  |  |  |  |  |  |
| CS | (0.8,0.4)  **0.61** | (0.0,0.0)  0.00 |  |  |  |  |  |  |  |  |  |  |  |  |  |  |  |  |  |
| SFT | (-0.1,-0.2)  -0.14 |  |  |  |  |  |  |  |  |  |  |  |  |  |  |  |  |  |  |

**Table S4 (pt.1 on this page; pt.2 on following page), related to Figure 4: Correlations between starting values and subsequent rates of change in our longitudinal studies.** Part 1 shows the correlations between starting measurements and the rate of change in either the ~4.5 mo following the first MRI, or in the ~4.5 mo to ~12 mo following MRI. Part 2 shows the correlations between starting measurements and the rate of change measured from the start to the end of the study — a ~4.5 mo period in Group YM, but ~12 mo period in Group MO. In addition to the variables analyzed in Tables S1-S3, we also checked for relationships between starting value for ln(age) and changes over time: Presuming a logarithmic growth curve, the youngest animals will tend to show the greatest changes over a fixed follow-up interval. Though within-group age differences are small at each time point, it’s important to check that a given variable’s predictive power is not merely due to initial age.

Correlation coefficients (Pearson’s r) are listed for each analysis as [(Group YM,Group MO) Combined]. Bold indicates significance (q < 0.05; FDR calculated on the total 600 tests in pts.1 and 2 of this Table): When r is listed for the combined analysis, group-specific r values are shown only for completeness. Where an * appears instead of an r value, an interaction between group and the correlation was suspected (*P* < 0.05) and statistics were run on each group separately. Combined analysis was not possible when testing for lagging predictors of change (i.e. dependent variable = change from ~4.5 mo to ~12 mo after study start) since only Group MO was tested over such a time period. Therefore only one number is shown for those analyses (right half of Table 4, pt.1). Note that Group YM’s r values appear in both pt.1 and pt.2 — since the “~4.5 mo later” time point was also the end of the study for those rats — but are combined with different Group MO data.

In pt.1, the rate of change in contrast sensitivity (‘CS’) — in the ~4.5 mo following the first MRI scan — is significantly correlated with (i.e., predicted by) the initial MRI measurements of retinal Mn^2+^ uptake: High Mn^2+^ uptake predicts substantial decline (negative r value) in that time period. That relationship is shown in main text Figure 4A. Interestingly, from ~4.5 to ~12 mo after that initial MRI scan, high initial Mn^2+^ uptake predicts relatively small *additional* declines in CS: During that period, rats that showed substantial declines from 0 to ~4.5 mo post-scan have plateaued at their new lower level of function. This pattern is depicted in Fig.S4. Because of this negative-than-later-positive relationship between Mn^2+^ uptake and subsequent changes in CS, those variables are poorly-correlated *when summing over the entire study period*, as in pt.2 of this table. In contrast, structural measures (e.g. retinal surface area) are not good predictors of CS changes over the time increments described in pt.1 of this table. However, when summing over the entire study period (pt.2), we find that structural measures can be significant predictors of CS declines. As shown in Fig.S4, the magnitude of the Mn^2+^-uptake-to-CS-change relationship appears much larger than the structure-to-CS-change relationship.

In pt.2, large sections of significant (bold) negative correlations are found for structural measurements. This indicates that rats which began the study with relatively large eye sizes showed the smallest subsequent increases in eye size — a convergence readily seen in Figs.S3 and S4. It also suggests that the structural measurements are tightly-linked with one-another — consistent with well-coordinated eye growth — with initial measurements of retinal surface area and lens thickness (for instance) being excellent predictors of growth in either structure.

| **Predicting Change in Measurement**  **(per unit ln(age))…** | from ~4.5 mo after study start to ~12 mo after study start  ***(Group MO)*** | CS | (-0.4) | (0.3) | (-0.2) | (-0.4) | (-0.4) | (-0.1) | (-0.4) | (-0.5) | (-0.3) | (-0.5) | (-0.2) | (0.2) | (-0.4) | (-0.4) | (-0.3) | (**0.6**) | (**0.7**) | (0.4) | (0.4) | (0.3) |  | (-0.2) | |
| --- | --- | --- | --- | --- | --- | --- | --- | --- | --- | --- | --- | --- | --- | --- | --- | --- | --- | --- | --- | --- | --- | --- | --- | --- | --- |
|  |  | SFT | (-0.1) | (0.2) | (0.0) | (-0.1) | (-0.2) | (0.0) | (-0.2) | (-0.2) | (-0.1) | (-0.2) | (-0.2) | (0.3) | (-0.1) | (-0.1) | (0.0) | (-0.1) | (-0.2) | (-0.1) | (-0.1) | (-0.1) |  | (-0.2) | |
|  |  | Body Weight | (-0.1) | (0.3) | (-0.4) | (-0.3) | (-0.3) | (-0.2) | (-0.4) | (-0.4) | (-0.3) | (-0.4) | (-0.2) | (0.3) | (-0.1) | (-0.1) | (0.1) | (0.0) | (-0.2) | (0.0) | (-0.2) | (-0.2) |  | (0.3) | |
|  | from the start of the study to ~4.5 mo later  ***(Group YM, Group MO) Combined*** | CS | (-0.8,-0.2)  **-0.46** | (0.0,0.2) 0.14 | (-0.8,-0.3)  **-0.51** | (-0.5,-0.1)  -0.26 | (**-0.8**,-0.1)  * | (0.4,0.1) 0.22 | (-0.8,-0.1)  -0.36 | (**-0.8**,0.0)  * | (-0.8,-0.2)  **-0.43** | (**-0.9**,-0.1)  * | (**-0.8**,0.0)  * | (0.5,0.0) 0.17 | (-0.7,-0.1)  -0.33 | (**-0.8**,-0.1)  * | (-0.6,-0.2)  -0.36 | (-0.6,-0.7)  **-0.68** | (-0.7,-0.8)  **-0.74** | (-0.6,-0.5)  **-0.51** | (-0.5,-0.6)  **-0.57** | (-0.4,-0.3)  -0.37 |  | (-0.6,0.1)  -0.19 | |
|  |  | SFT | (-0.2,-0.2)  -0.23 | (0.2,-0.5)  -0.18 | (0.2,-0.3)  -0.09 | (-0.4,0.0)  -0.18 | (-0.1,-0.1)  -0.11 | (-0.2,-0.3)  -0.25 | (-0.2,-0.2)  -0.19 | (0.0,-0.2)  -0.09 | (-0.1,-0.2)  -0.18 | (0.0,-0.1)  -0.07 | (0.1,0.1) 0.11 | (-0.2,-0.1)  -0.13 | (0.0,0.0)  -0.03 | (0.1,0.0) 0.01 | (-0.2,-0.2)  -0.18 | (-0.1,0.1) 0.05 | (-0.1,0.2) 0.11 | (0.1,0.1) 0.07 | (-0.2,0.3) 0.08 | (0.0,0.3) 0.16 |  | (-0.3,-0.2)  -0.29 | |
|  |  | Body Weight | (**-0.9**,0.0)  ***** | (0.0,0.0) 0.03 | (-0.7,-0.2)  -0.37 | (-0.5,-0.3)  -0.37 | (-0.8,-0.3)  **-0.50** | (0.2,0.2) 0.18 | (-0.8,-0.2)  **-0.44** | (-0.6,-0.3)  **-0.44** | (-0.7,-0.3)  **-0.46** | (-0.8,-0.3)  **-0.49** | (-0.6,-0.4)  **-0.45** | (0.6,0.0) 0.23 | (-0.7,-0.3)  **-0.49** | (-0.7,-0.3)  **-0.49** | (-0.6,-0.2)  -0.38 | (**-0.8**,0.4)  * | (**-0.8**,0.2)  * | (-0.6,**0.7**)  * | (-0.7,-0.4)  **-0.53** | (-0.6,-0.5)  **-0.57** |  | (-0.4,0.5)  * | |
|  |  |  | Body Weight | SFT | CS | Ant. Chamber Depth | Lens Thickness | Vit. Chamber Depth | Axial Length | Corneal rC | Anterior Lens rC | Posterior Lens rC | Refractive State | Retinal Thickness | Retinal Extent | Retinal Surface Area | Retinal Volume | ΔR_1_  (inner retina) | ΔR_1,Dark_  (outer retina) | ΔR_1,Light_  (outer retina) | ΔR_1,Dark_ - ΔR_1,Light_ (outer retina) | ΔR_1,Dark_ / ΔR_1,Light_ (outer retina) |  | ln(Age) | |
|  |  |  | **Measured at the Start of the Study** | | | | | | | | | | | | | | | | | | | | | |  |

| **Predicting Change in Measurement (per unit ln(age))…** | from the start to the end of study  ***(Group YM spanning ~4.5 mo, Group MO spanning ~12 mo)*** | ΔR_1,Dark_ / ΔR_1,Light_  (outer retina) | (-0.3,0.1)  -0.10 | (-0.2,0.1)  -0.06 | (0.0,-0.6)  -0.31 | (0.0,0.4) 0.21 | (0.1,-0.1) 0.02 | (0.1,-0.2)  -0.09 | (0.1,0.0) 0.03 | (0.3,0.0)  0.15 | (0.1,0.1)  0.07 | (0.1,0.0)  0.06 | (0.2,0.2)  0.23 | (0.0,-0.5)  -0.24 | (0.1,0.0)  0.04 | (0.1,0.2)  0.14 | (0.0,-0.1)  -0.05 | (-0.4,-0.1)  -0.26 | (-0.4,-0.5)  -0.48 | (-0.3,0.1)  -0.11 | (-0.4,-0.6)  **-0.54** | (-0.5,-0.7)  **-0.61** |  | (0.4,0.1)  0.27 |
| --- | --- | --- | --- | --- | --- | --- | --- | --- | --- | --- | --- | --- | --- | --- | --- | --- | --- | --- | --- | --- | --- | --- | --- | --- |
|  |  | ΔR_1,Dark_ - ΔR_1,Light_  (outer retina) | (-0.2,-0.1) -0.14 | (-0.3,-0.1) -0.20 | (0.1,-0.3) -0.13 | (-0.1,0.4) 0.13 | (0.1,-0.1) 0.00 | (0.0,-0.6) -0.28 | (0.1,-0.2) -0.04 | (0.3,-0.1) 0.13 | (0.0,0.0) 0.00 | (0.1,0.1) 0.06 | (0.3,0.4) 0.36 | (-0.2,-0.5) -0.37 | (0.1,0.0) 0.04 | (0.2,0.2) 0.16 | (-0.1,-0.2) -0.13 | (-0.4,-0.4) -0.37 | (-0.4,-0.6) -0.50 | (-0.4,-0.3) -0.35 | (-0.3,-0.4) -0.33 | (-0.1,-0.3) -0.23 |  | (0.5,-0.3) 0.07 |
|  |  | ΔR_1,Light_  (outer retina) | (-0.5,0.2)  -0.14 | (-0.3,0.6)  * | (-0.6,-0.1)  -0.33 | (-0.5,0.0)  -0.24 | (**-0.8**,0.1)  * | (0.0,-0.4)  -0.18 | (-0.8,0.0)  -0.41 | (-0.7,0.1)  -0.31 | (-0.7,0.0)  -0.38 | (-0.8,-0.1)  -0.48 | (-0.6,0.4)  * | (0.3,0.1) 0.19 | (**-0.7**,0.2)  * | (**-0.8**,0.2)  * | (**-0.7**,0.4)  * | (-0.5,-0.2)  -0.36 | (-0.4,-0.3)  -0.35 | (-0.7,0.0)  -0.34 | (-0.1,-0.3)  -0.18 | (0.2,-0.1) 0.07 |  | (-0.3,0.1)  -0.11 |
|  |  | ΔR_1,Dark_  (outer retina) | (-0.5,0.1)  -0.22 | (-0.6,0.4)  * | (-0.3,-0.3)  -0.34 | (-0.5,0.3)  -0.08 | (-0.5,0.0)  -0.24 | (0.0,-0.7)  -0.35 | (-0.5,-0.1)  -0.32 | (-0.2,0.0)  -0.08 | (-0.5,0.0)  -0.26 | (-0.5,-0.1)  -0.28 | (-0.1,0.6)  0.25 | (0.0,-0.3)  -0.17 | (-0.4,0.1)  -0.15 | (-0.4,0.3)  -0.05 | (-0.6,0.1)  -0.22 | (-0.7,-0.4)  **-0.58** | (-0.7,-0.7)  **-0.67** | (-0.8,-0.3)  **-0.54** | (-0.3,-0.5)  -0.41 | (0.0,-0.3)  -0.14 |  | (0.2,-0.2)  0.03 |
|  |  | ΔR_1_,  (inner retina) | (-0.6,0.2)  -0.24 | (-0.4,0.3)  -0.01 | (-0.5,0.1)  -0.21 | (-0.6,0.1)  -0.22 | (**-0.8**,0.2)  * | (0.2,-0.3)  -0.06 | (**-0.8**,0.1)  * | (-0.6,0.2)  * | (**-0.8**,0.1)  * | (**-0.9**,0.2)  * | (-0.6,0.5)  * | (0.3,-0.3)  0.02 | (**-0.8**,0.2)  * | (**-0.8**,0.3)  * | (**-0.8**,0.2)  * | (-0.8,-0.8)  **-0.80** | (-0.7,-0.8)  **-0.74** | (-0.8,-0.6)  **-0.72** | (-0.4,-0.3)  -0.33 | (0.0,0.0)  -0.02 |  | (-0.2,-0.4)  -0.29 |
|  |  | Retinal Volume | (-0.7,-0.5)  **-0.59** | (0.1,0.7) 0.43 | (-0.1,0.0)  -0.06 | (-0.4,-0.6)  **-0.53** | (-0.7,-0.7)  **-0.71** | (0.1,-0.2)  -0.09 | (-0.7,-0.7)  **-0.71** | (-0.7,-0.7)  **-0.69** | (-0.7,-0.6)  **-0.62** | (-0.7,-0.7)  **-0.70** | (-0.6,-0.3)  -0.43 | (0.4,0.0) 0.16 | (-0.6,-0.5)  **-0.57** | (-0.6,-0.5)  **-0.54** | (-0.7,-0.5)  **-0.57** | (-0.5,0.1)  -0.15 | (-0.4,0.1)  -0.14 | (-0.4,0.2)  -0.06 | (-0.4,-0.1)  -0.21 | (-0.1,-0.2)  -0.14 |  | (-0.8,-0.3)  **-0.53** |
|  |  | Retinal Surface Area | (-0.5,-0.8)  **-0.68** | (-0.1,0.5) 0.25 | (-0.7,0.0)  -0.31 | (-0.7,-0.8)  **-0.73** | (-0.8,-0.9)  **-0.82** | (0.1,0.0) 0.03 | (-0.8,-0.8)  **-0.79** | (-0.6,-0.8)  **-0.73** | (-0.7,-0.8)  **-0.76** | (-0.7,-0.8)  **-0.75** | (-0.5,-0.6)  **-0.55** | (0.4,0.2) 0.28 | (-0.7,-0.9)  **-0.80** | (-0.7,-0.9)  **-0.82** | (-0.5,-0.8)  **-0.68** | (-0.6,0.2)  -0.18 | (-0.5,0.3)  * | (-0.6,0.3)  * | (-0.4,0.0)  -0.13 | (-0.2,0.0)  -0.10 |  | (-0.1,-0.1)  -0.07 |
|  |  | Retinal Extent | (-0.7,-0.9)  **-0.81** | (-0.1,0.6) 0.30 | (-0.6,-0.1)  -0.32 | (-0.8,-0.7)  **-0.76** | (-0.9,-0.9)  **-0.91** | (0.0,-0.1)  -0.03 | (-0.9,-0.9)  **-0.89** | (-0.7,-0.9)  **-0.81** | (-0.8,-0.9)  **-0.84** | (-0.8,-0.8)  **-0.84** | (-0.6,-0.6)  **-0.57** | (0.5,0.2) 0.34 | (-0.9,-0.9)  **-0.88** | (-0.8,-0.9)  **-0.86** | (-0.6,-0.8)  **-0.73** | (**-0.8**,0.1)  * | (**-0.7**,0.2)  * | (-0.7,0.3)  * | (-0.6,0.0)  -0.26 | (-0.4,-0.1)  -0.2 |  | (-0.2,-0.1)  -0.18 |
|  |  | Retinal Thickness | (-0.1,-0.2)  -0.19 | (0.3,0.4) 0.35 | (0.1,0.0) 0.08 | (0.1,-0.1)  -0.01 | (-0.1,-0.4)  -0.25 | (-0.2,-0.6)  -0.43 | (-0.1,-0.5)  -0.31 | (-0.2,-0.3)  -0.29 | (-0.1,-0.3)  -0.21 | (-0.1,-0.4)  -0.29 | (-0.1,0.4) 0.15 | (-0.2,-0.6)  -0.42 | (0.1,-0.1)  -0.05 | (0.1,0.0) 0.01 | (-0.2,-0.3)  -0.24 | (0.1,0.0) 0.05 | (0.1,0.0) 0.01 | (0.0,0.2) 0.13 | (0.1,-0.3)  -0.09 | (0.3,-0.3)  -0.05 |  | (-0.6,-0.4)  **-0.51** |
|  |  | Refractive State | (-0.5,-0.3)  -0.37 | (0.2,0.2) 0.21 | (-0.3,0.1)  -0.06 | (-0.2,-0.2)  -0.21 | (-0.7,-0.3)  -0.46 | (0.5,0.4) 0.41 | (-0.6,-0.1)  -0.34 | (-0.8,-0.3)  **-0.54** | (-0.7,-0.2)  -0.41 | (-0.7,-0.1)  -0.39 | (-0.9,-0.6)  **-0.72** | (0.6,0.6) **0.59** | (-0.7,-0.4)  **-0.56** | (-0.8,-0.4)  **-0.58** | (-0.4,-0.2)  -0.28 | (-0.4,0.1)  -0.13 | (-0.5,0.0)  -0.19 | (-0.4,0.0)  -0.18 | (-0.5,0.1)  -0.17 | (-0.4,0.1)  -0.11 |  | (-0.6,0.0)  -0.25 |
|  |  | Posterior Lens rC | (-0.9,-0.5)  **-0.71** | (-0.2,0.5) 0.20 | (-0.5,-0.2)  -0.35 | (-0.7,-0.6)  **-0.60** | (-1.0,-0.8)  **-0.88** | (0.1,-0.2) -0.05 | (-0.9,-0.8)  **-0.85** | (-0.8,-0.8)  **-0.80** | (-0.9,-0.8)  **-0.82** | (-1.0,-0.9)  **-0.93** | (-0.7,-0.5)  **-0.56** | (0.5,0.2) 0.33 | (-0.9,-0.5)  **-0.69** | (-0.9,-0.5)  **-0.67** | (-0.8,-0.4)  **-0.59** | (**-0.9**,0.4)  * | (**-0.9**,0.4)  * | (**-0.8**,**0.6**)  * | (-0.7,-0.2)  -0.41 | (-0.4,-0.3)  -0.33 |  | (-0.5,0.1)  -0.13 |
|  |  | Anterior Lens rC | (-0.9,-0.5)  **-0.67** | (-0.3,0.1)  -0.05 | (-0.4,-0.3)  -0.30 | (-0.8,-0.4)  **-0.54** | (-1.0,-0.7)  **-0.81** | (0.0,-0.1)  -0.09 | (-1.0,-0.6)  **-0.79** | (-0.8,-0.7)  **-0.77** | (-0.9,-0.8)  **-0.84** | (-0.9,-0.7)  **-0.81** | (-0.7,-0.5)  **-0.55** | (0.6,0.1) 0.30 | (-0.9,-0.5)  **-0.70** | (-0.9,-0.5)  **-0.68** | (-0.8,-0.5)  **-0.63** | (**-0.8**,0.1)  * | (**-0.8**,0.1)  * | (**-0.8**,0.4)  * | (-0.6,-0.2)  -0.42 | (-0.3,-0.2)  -0.23 |  | (-0.5,0.2)  -0.09 |
|  |  | Corneal rC | (-0.8,-0.8)  **-0.77** | (-0.1,0.4) 0.19 | (-0.5,-0.3)  -0.37 | (-0.4,-0.7)  **-0.57** | (-0.9,-0.9)  **-0.92** | (0.2,-0.2) -0.03 | (-0.8,-0.9)  **-0.88** | (-1.0,-1.0)  **-0.96** | (-0.9,-0.9)  **-0.89** | (-0.9,-0.9)  **-0.89** | (-0.9,-0.6)  **-0.72** | (0.7,0.2) 0.42 | (-0.9,-0.8)  **-0.83** | (-0.9,-0.8)  **-0.83** | (-0.6,-0.7)  **-0.67** | (-0.7,0.2)  * | (**-0.7**,0.2)  * | (-0.7,0.3)  * | (-0.6,-0.1)  -0.30 | (-0.4,-0.1)  -0.21 |  | (-0.6,0.0)  -0.29 |
|  |  | Axial Length | (-0.9,-0.7)  **-0.77** | (-0.3,0.5) 0.14 | (-0.5,-0.4)  -0.43 | (-0.8,-0.7)  **-0.72** | (-0.9,-0.9)  **-0.91** | (0.0,-0.3) -0.16 | (-0.9,-0.9)  **-0.92** | (-0.7,-0.9)  **-0.81** | (-0.9,-0.9)  **-0.88** | (-0.9,-0.9)  **-0.93** | (-0.6,-0.4)  -0.47 | (0.5,0.0) 0.22 | (-0.9,-0.7)  **-0.76** | (-0.9,-0.6)  **-0.73** | (-0.8,-0.6)  **-0.70** | (**-0.9**,0.3)  * | (**-0.9**,0.3)  * | (**-0.9**,0.5)  * | (-0.6,-0.1)  -0.36 | (-0.3,-0.2)  -0.27 |  | (-0.3,0.0)  -0.09 |
|  |  | Vit. Chamber Depth | (-0.3,-0.2)  -0.28 | (-0.6,-0.2)  -0.38 | (-0.2,-0.3)  -0.28 | (-0.1,-0.3)  -0.21 | (-0.2,-0.3)  -0.27 | (-0.4,-0.3)  -0.36 | (-0.2,-0.4)  -0.33 | (-0.1,-0.3)  -0.25 | (-0.2,-0.4)  -0.33 | (-0.2,-0.4)  -0.30 | (0.0,0.0) 0.00 | (0.2,-0.3)  -0.07 | (-0.2,-0.1)  -0.16 | (-0.2,-0.2)  -0.16 | (-0.3,-0.3)  -0.30 | (-0.3,0.0)  -0.14 | (-0.3,0.1)  -0.08 | (-0.5,0.2)  -0.11 | (-0.1,0.0)  -0.05 | (0.1,-0.1) 0.01 |  | (-0.1,0.1) 0.04 |
|  |  | Lens Thickness | (-0.9,-0.7)  **-0.80** | (-0.2,**0.6**)  * | (-0.5,-0.3)  -0.39 | (-0.7,-0.6)  **-0.63** | (-1.0,-0.9)  **-0.94** | (0.2,-0.3)  -0.08 | (-0.9,-0.9)  **-0.91** | (-0.8,-0.9)  **-0.83** | (-0.9,-0.8)  **-0.86** | (-0.9,-0.9)  **-0.93** | (-0.7,-0.4)  **-0.53** | (0.5,0.1) 0.30 | (-0.9,-0.7)  **-0.81** | (-0.9,-0.7)  **-0.76** | (-0.7,-0.6)  **-0.67** | (**-0.9**,0.3)  * | (**-0.9**,0.2)  * | (**-0.8**,0.5)  * | (-0.7,-0.2)  -0.42 | (-0.4,-0.3)  -0.35 |  | (-0.3,-0.1) -0.18 |
|  |  | Ant. Chamber Depth | (-0.6,-0.5)  **-0.55** | (-0.2,0.5) 0.21 | (-0.4,-0.2)  -0.27 | (-0.8,-0.9)  **-0.84** | (-0.7,-0.7)  **-0.71** | (0.0,-0.1)  -0.08 | (-0.7,-0.8)  **-0.75** | (-0.4,-0.7)  **-0.59** | (-0.7,-0.8)  **-0.73** | (-0.6,-0.9)  **-0.75** | (-0.3,-0.4)  -0.37 | (0.1,0.2) 0.16 | (-0.7,-0.6)  **-0.63** | (-0.6,-0.6)  **-0.62** | (-0.7,-0.5)  **-0.58** | (-0.7,0.3)  * | (-0.6,0.5)  * | (-0.6,0.5)  * | (-0.4,0.0)  -0.16 | (-0.2,0.0)  -0.10 |  | (0.0,0.1) 0.09 |
|  |  | CS | (-0.8,-0.2)  **-0.49** | (0.0,0.4) 0.23 | (-0.8,-0.6)  **-0.67** | (-0.5,-0.4)  -0.43 | (-0.8,-0.4)  **-0.59** | (0.4,0.0) 0.17 | (-0.8,-0.4)  **-0.56** | (-0.8,-0.3)  **-0.53** | (-0.8,-0.3)  **-0.52** | (-0.9,-0.4)  **-0.61** | (-0.8,-0.1)  -0.43 | (0.5,0.0) 0.22 | (-0.7,-0.4)  **-0.53** | (-0.8,-0.4)  **-0.56** | (-0.6,-0.4)  **-0.50** | (-0.6,-0.1) -0.35 | (-0.7,-0.1) -0.37 | (-0.6,0.0) -0.26 | (-0.5,-0.1) -0.30 | (-0.4,-0.1) -0.22 |  | (-0.6,-0.1) -0.33 |
|  |  | SFT | (-0.2,-0.1) -0.17 | (0.2,-0.1) 0.05 | (0.2,0.1) 0.15 | (-0.4,-0.1) -0.22 | (-0.1,-0.1) -0.10 | (-0.2,-0.2) -0.19 | (-0.2,-0.2) -0.16 | (0.0,-0.2) -0.10 | (-0.1,-0.2) -0.15 | (0.0,-0.1) -0.08 | (0.1,0.0) 0.04 | (-0.2,0.1) -0.03 | (0.0,0.0)  -0.03 | (0.1,0.0) 0.03 | (-0.2,0.0) -0.07 | (-0.1,-0.2) -0.16 | (-0.1,0.0) -0.03 | (0.1,-0.4) -0.21 | (-0.2,0.4) 0.14 | (0.0,0.4) 0.22 |  | (-0.4,-0.4) -0.38 |
|  |  | Body Weight | (-0.9,-0.2)  **-0.54** | (0.0,0.4) 0.20 | (-0.7,-0.4)  **-0.55** | (-0.5,-0.5)  **-0.51** | (-0.8,-0.5)  **-0.66** | (0.2,0.0) 0.07 | (-0.8,-0.5)  **-0.64** | (-0.6,-0.6)  **-0.62** | (-0.7,-0.5)  **-0.63** | (-0.8,-0.6)  **-0.70** | (-0.6,-0.4)  **-0.49** | (0.6,0.3) 0.44 | (-0.7,-0.3)  **-0.53** | (-0.7,-0.4)  **-0.54** | (-0.6,-0.1)  -0.34 | (**-0.8**,0.1)  * | (-0.8,-0.1)  -0.43 | (-0.6,0.3)  * | (-0.7,-0.4)  **-0.55** | (-0.6,-0.4)  **-0.50** |  | (-0.4,0.4)  * |
|  |  |  | Body Weight | SFT | CS | Ant. Chamber Depth | Lens Thickness | Vit. Chamber Depth | Axial Length | Corneal rC | Anterior Lens rC | Posterior Lens rC | Refractive State | Retinal Thickness | Retinal Extent | Retinal Surface Area | Retinal Volume | ΔR_1_  (inner retina) | ΔR_1,Dark_  (outer retina) | ΔR_1,Light_  (outer retina) | ΔR_1,Dark_ - ΔR_1,Light_ (outer retina) | ΔR_1,Dark_ / ΔR_1,Light_ (outer retina) |  | ln(Age) |
|  |  |  | **Measured at the Start of the Study** | | | | | | | | | | | | | | | | | | | | | |


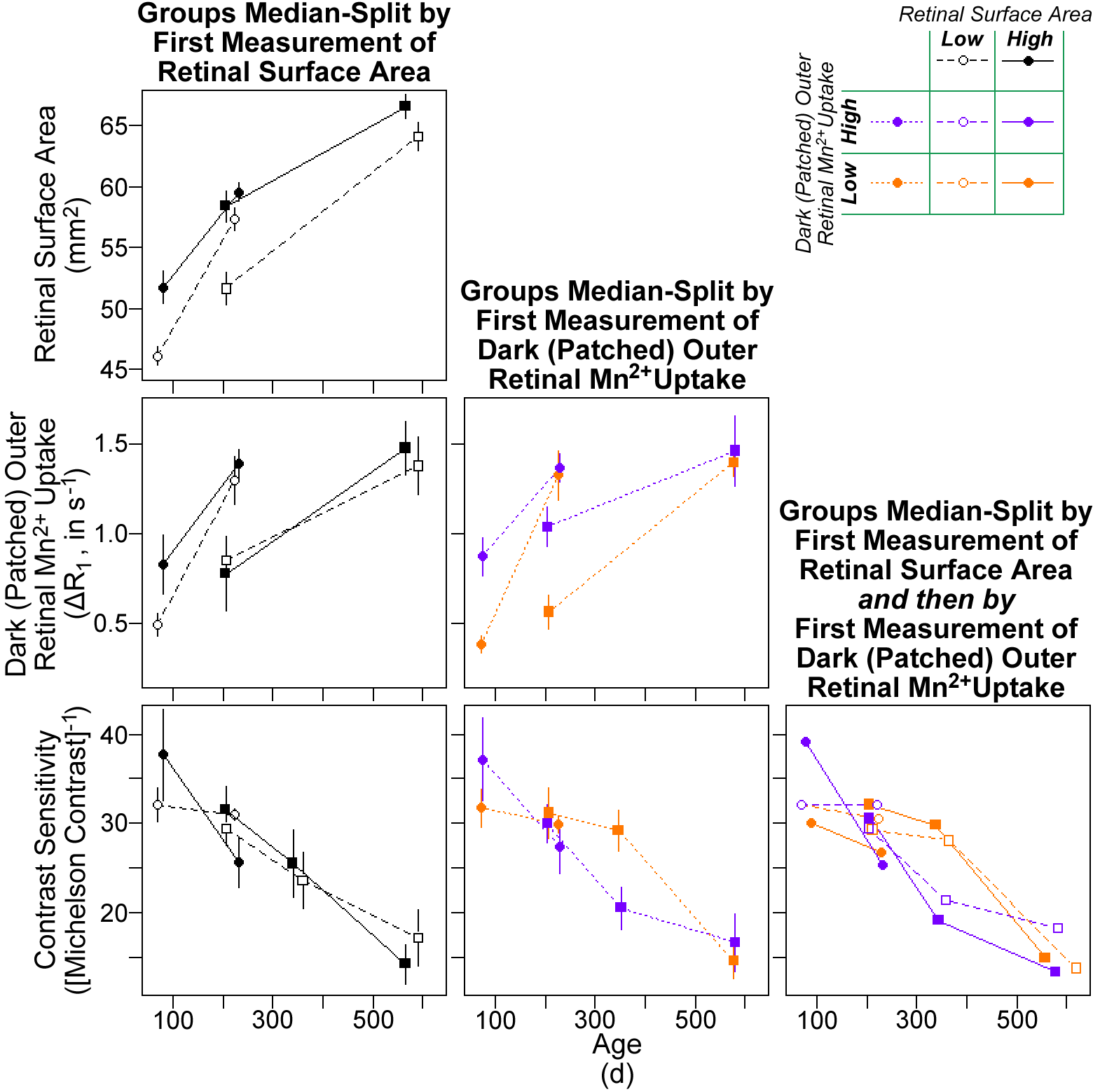


**Figure S4, related to Figure 4: Visualization of regression results linking initial Mn^2+^ uptake (and optics / eye size) measurements to subsequent CS declines.**


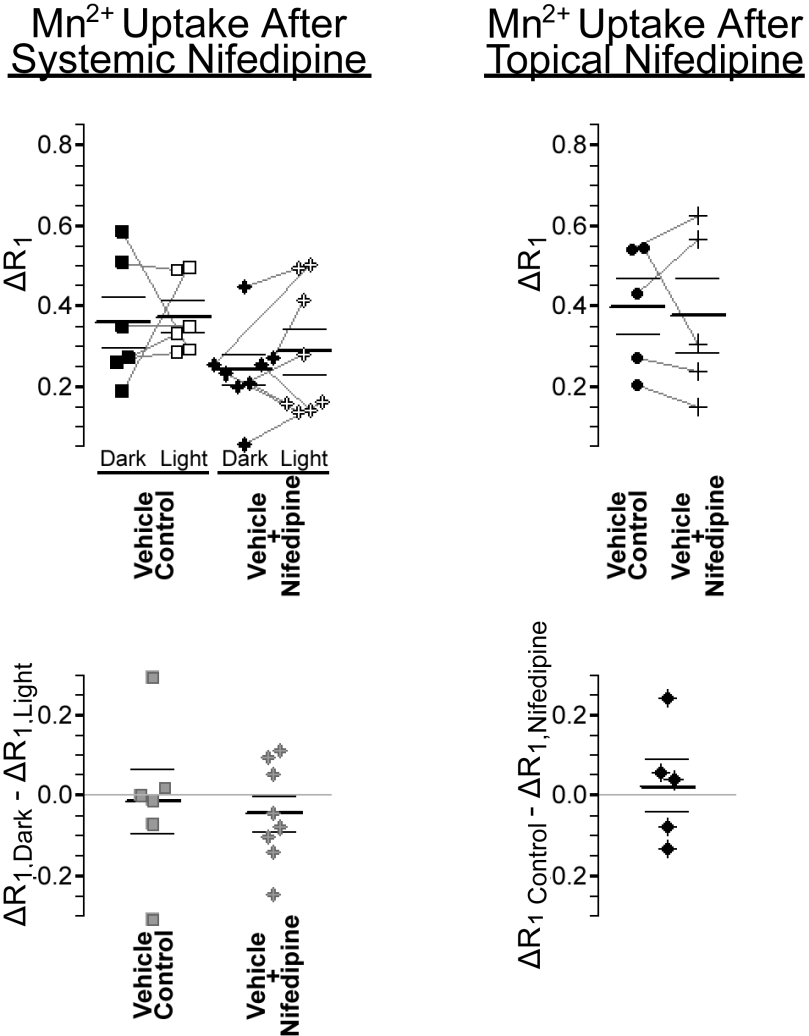


**Figure S5, related to Figure 5: *Inner* retinal Mn^2+^ uptake (ΔR_1_; in s^-1^) in nifedipine-treated and vehicle control eyes.**


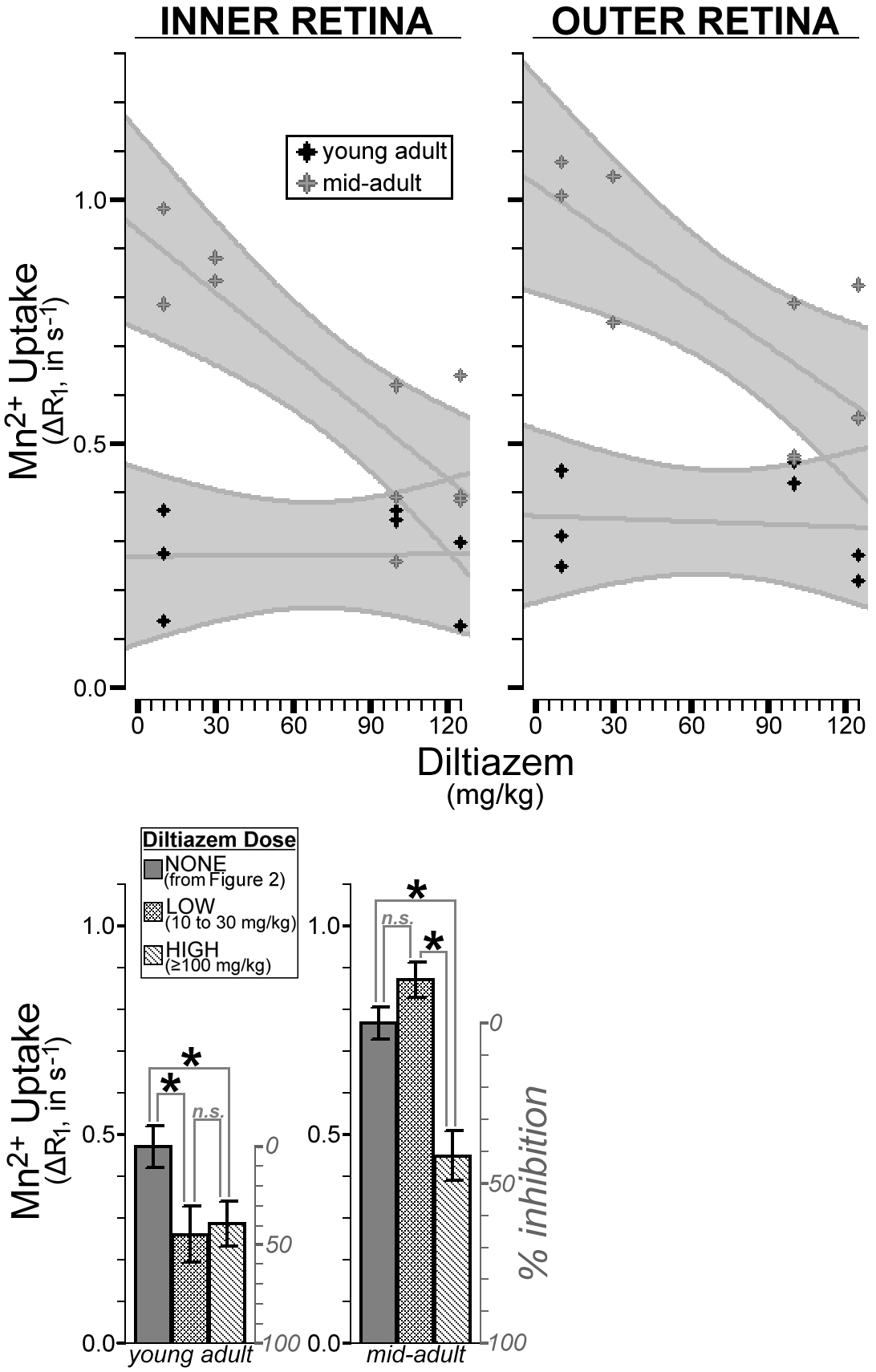


**Figure S6, related to Figure 7: Inner and outer retinal data show an age-related change in sensitivity to diltiazem.**

**Supplemental References**

1. Douglas RM, Alam NM, Silver BD, McGill TJ, Tschetter et al. (2005) Independent visual threshold measurements in the two eyes of freely moving rats and mice using a virtual-reality optokinetic system. Visual Neuroscience 22: 677-684.

2. Prusky GT, Silver BD, Tschetter WW, Alam NM, Douglas RM (2008) Experience-dependent plasticity from eye opening enables lasting, visual cortex-dependent enhancement of motion vision. The Journal of Neuroscience 28: 9817-9827.

3. Berkowitz BA, Roberts R, Luan H, Peysakhov J, Mao X, et al. (2004) Dynamic contrast-enhanced MRI measurements of passive permeability through blood retinal barrier in diabetic rats. Investigative Ophthalmology & Visual Science 45: 2391-2398.

4. Chui TY, Bissig D, Berkowitz BA, Akula JD (2012) Refractive development in the "ROP rat". Journal of Ophthalmology 2012: 956705.

5. Hughes A (1979) A schematic eye for the rat. Vision Research 19: 569-588.

6. Srinivasan VJ, Wojtkowski M, Fujimoto JG, Duker JS (2006) In vivo measurement of retinal physiology with high-speed ultrahigh-resolution optical coherence tomography. Optics Letters 31: 2308-2310.

7. Srinivasan VJ, Ko TH, Wojtkowski M, Carvalho M, Clermont A, et al. (2006) Noninvasive volumetric imaging and morphometry of the rodent retina with high-speed, ultrahigh-resolution optical coherence tomography. Investigative Ophthalmology & Visual Science 47: 5522-5528.

8. Hariri S, Moayed AA, Dracopoulos A, Hyun C, Boyd S, et al. (2009) Limiting factors to the OCT axial resolution for in-vivo imaging of human and rodent retina in the 1060 nm wavelength range. Optics Express 17: 24304-24316.

9. Hagins WA, Penn RD, Yoshikami S (1970) Dark current and photocurrent in retinal rods. Biophysical Journal 10: 380-412.

10. Katz ML, Kutryb MJ, Norberg M, Gao CL, White RH, et al. (1991) Maintenance of opsin density in photoreceptor outer segments of retinoid-deprived rats. Investigative Ophthalmology & Visual Science 32: 1968-1980.

11. Cohen AI (1971) Electron microscope observations on form changes in photoreceptor outer segments and their saccules in response to osmotic stress. Journal of Cell Biology 48: 547-565.

12. Cunea A., Jeffery G. (2007) The ageing photoreceptor. Visual Neuroscience 24: 151-155.

13. Case CP, Plummer CJ (1993) Changing the light intensity of the visual environment results in large differences in numbers of synapses and in photoreceptor size in the retina of the young adult rat. Neuroscience 55: 653-666.

14. Penn JS, Williams TP (1986) Photostasis: Regulation of daily photon-catch by rat retinas in response to various cyclic illuminances. Experimental Eye Research 43: 915-928.

15. Massof RW, Chang FW (1972) A revision of the rat schematic eye. Vision Research 12: 793-796.

16. Wiesenfeld Z, Branchek T (1976) Refractive state and visual acuity in the hooded rat. Vision Research 16: 823-827.

17. Hughes A (1977) The refractive state of the rat eye. Vision Research 17: 927-939.

18. Katz ML, Robison Jr WG (1986) Evidence of cell loss from the rat retina during senescence. Experimental Eye Research 42: 293-304.

19. Feng L, Sun Z, Han H, Zhou Y, Zhang M (2007) No age-related cell loss in three retinal nuclear layers of the Long-Evans rat. Visual Neuroscience 24: 799-803.

20. Mansour H, Chamberlain CG, Weible II MW, Hughes S, Chu Y, Chan-Ling T (2008) Aging-related changes in astrocytes in the rat retina: imbalance between cell proliferation and cell death reduces astrocyte availability. Aging Cell 7: 526-540.

21. Harman AM, MacDonald A, Meyer P, Ahmat A (2003) Numbers of neurons in the retinal ganglion cell layer of the rat do not change throughout life. Gerontology 49: 350-355.

22. Berkowitz BA, Roberts R, Goebel DJ, Luan H (2006) Noninvasive and simultaneous imaging of layer-specific retinal functional adaptation by manganese-enhanced MRI. Investigative Ophthalmology & Visual Science 47: 2668-2674.

23. Tofts PS, Porchia A, Jin Y, Roberts R, Berkowitz BA (2010) Toward clinical application of manganese-enhanced MRI of retinal function. Brain Research Bulletin 81: 333-338.

24. Bissig D, Berkowitz BA (2011) Same-session functional assessment of rat retina and brain with manganese-enhanced MRI. NeuroImage 58: 749-760.

25. Berkowitz BA, Roberts R, Penn JS, Gradianu M (2007) High-resolution manganese-enhanced MRI of experimental retinopathy of prematurity. Investigative Ophthalmology & Visual Science 48: 4733-4740.
